# Substrate‐dependent cluster density dynamics of *Corynebacterium glutamicum* phosphotransferase system permeases

**DOI:** 10.1111/mmi.14224

**Published:** 2019-03-18

**Authors:** Gustavo Benevides Martins, Giacomo Giacomelli, Oliver Goldbeck, Gerd M. Seibold, Marc Bramkamp

**Affiliations:** ^1^ Faculty of Biology Ludwig‐Maximilians‐Universität München Großhaderner Straße 2‐4 Planegg‐Martinsried 82152 Germany; ^2^ Institute of Microbiology and Biotechnology Ulm University Albert‐Einstein Allee 11 Ulm 89081 Germany

## Abstract

Many bacteria take up carbohydrates by membrane‐integral sugar specific phosphoenolpyruvate‐dependent carbohydrate:phosphotransferase systems (PTS). Although the PTS is centrally involved in regulation of carbon metabolism in different bacteria, little is known about localization and putative oligomerization of the permease subunits (EII). Here, we analyzed localization of the fructose specific PtsF and the glucose specific PtsG transporters, as well as the general components EI and HPr from *Corynebacterium glutamicum* using widefield and single molecule localization microscopy. PtsF and PtsG form membrane embedded clusters that localize in a punctate pattern. Size, number and fluorescence of the membrane clusters change upon presence or absence of the transported substrate, and a direct influence of EI and HPr was not observed. In presence of the transport substrate, EII clusters significantly increased in size. Photo‐activated localization microscopy data revealed that, in presence of different carbon sources, the number of EII proteins per cluster remains the same, however, the density of these clusters reduces. Our work reveals a simple mechanism for efficient membrane occupancy regulation. Clusters of PTS EII transporters are densely packed in absence of a suitable substrate. In presence of a transported substrate, the EII proteins in individual clusters occupy larger membrane areas.

## Graphical abstract

PTS permeases cluster in substrate dependent manner in the membrane of *Corynebacterium glutamicum. *In absence of the transport substrate (here glucose) the PTS permease forms densely packed clusters. In presence of the correct sugar the permease proteins rearrange and cover a larger membrane area.

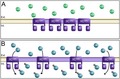

## Introduction

**Figure 1:**
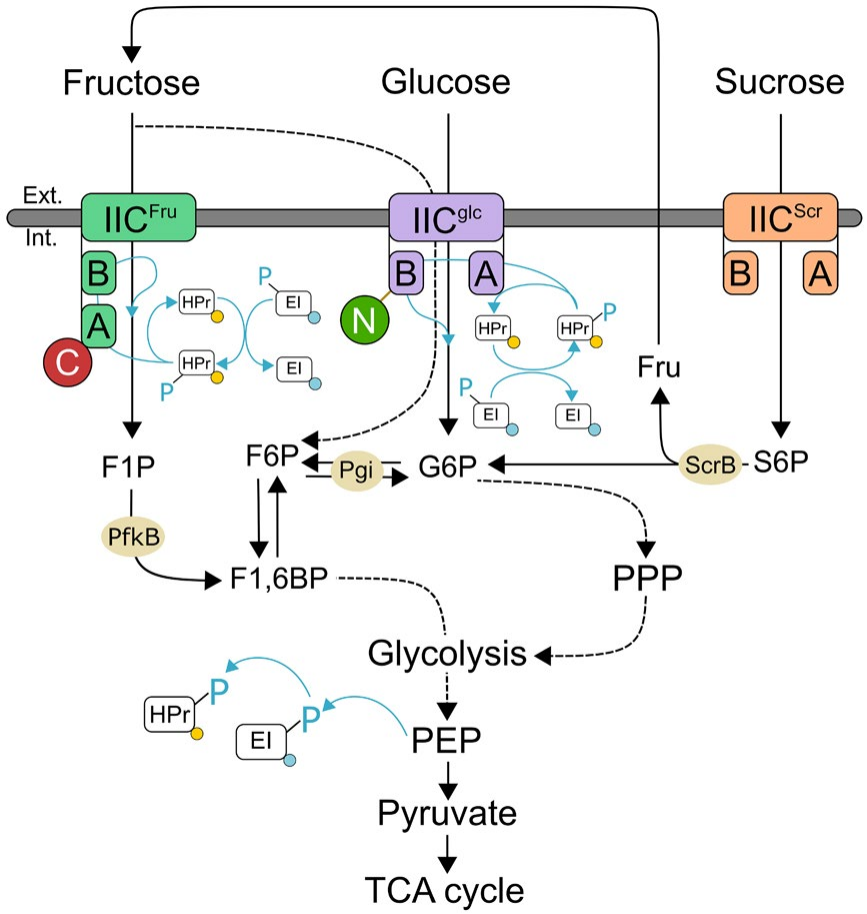
Schematic view of the sugar uptake mediated by the phosphoenolpyruvate‐dependent phosphotransferase systems (PTS) in *Corynebacterium glutamicum*. Blue lines indicate phosphoryl group (PO_3_^2−^) transfer. Fluorescent proteins added via allelic replacement represented as C for mCherry in subunit EIIA of PtsF, N for mNeonGreen‐Linker in subunit EIIB of PtsG, yellow circles of mVenus attached to HPr and blue circles or eCFP attached to EI. F1P *fructose‐1‐phosphate*, F6P *fructose‐6‐phosphate*, F1,6BP *fructose‐1,6‐biphosphate*, G6P *glucose‐6‐phosphate*, Fru *fructose*, S6P *sucrose‐6‐phosphate*, PPP *Pentose phosphate pathway*, PEP *phosphoenolpyruvate*, scrB *beta‐fructofuranosidase* (putative S6P hydrolase), pgi *glucose‐6‐phosphate isomerase*, PfkB *1‐phosphofructokinase*.

In heterotrophic bacteria, uptake of suitable carbohydrates is an essential task for the cells in their quest for food and, hence, subject to meticulous regulation. In growth media with several carbon sources, many bacteria use preferred sugars, such as glucose first, which leads to the well‐known diauxic growth behavior (Epps and Gale, 1942; Monod, 1942). Regulation of sequential carbohydrate usage and transport of carbohydrates is often governed by enzyme complexes termed phosphoenolpyruvate‐dependent carbohydrate: phosphotransferase systems (PTS) (Lengeler, 2000; Deutscher *et al.*, 2006; Gorke and Stulke, 2008; Lengeler and Jahreis, 2009; Deutscher *et al.*, 2014; Lengeler, 2015). Interestingly, some bacteria are known to co‐ferment different carbohydrates, with *Corynebacterium glutamicum* being a prominent example (Wendisch *et al.*, 2000; Moon *et al.*, 2007). *C. glutamicum* is a facultative anaerobic, chemoheterotroph that is used in the large scale industrial production of amino acids (Wendisch, 2017; Becker *et al.*, 2018; Shah *et al.*, 2018). In industrial applications, the main feedstock used in most of the established fermentation processes are molasses and starch hydrolysates, which contain a broad spectrum of simple carbohydrates, but mostly glucose, fructose and sucrose. These sugars are taken up and phosphorylated during transport into the cell via PTS. The PTS consists of two common energy‐coupling cytoplasmic proteins, enzyme I (EI) and histidine‐containing phosphocarrier protein protein (HPr) (Kuhlmann *et al.*, 2015), which are encoded by *ptsI* (cg2117) and *ptsH* (cg2121), respectively (Fig. 1), and a series of sugar‐specific enzyme II (EII) complexes located in the membrane. The EII complexes are typically divided into three protein domains, EIIA, EIIB and EIIC, whose organization differs between permeases and organisms, ranging from setups in which the three EII domains are fused in a single protein to a variety of differently fused and unfused domains (Gorke and Stulke, 2008; Deutscher *et al.*, 2014). The passage of specific sugars through the membrane is catalyzed by the transmembrane EIIC part of the system (Neidhardt and Curtiss, 1996). Structural analysis of EIIC subunits revealed that they likely work as dimers. They contain a substrate recognition and a transport part (Cao *et al.*, 2011). The PTS couple transports and converts carbohydrates into their respective phosphoesters using the energy of phosphoryl group translocation. The phosphoryl group from phosphoenolpyruvate (PEP) is transferred to EI, HPr, EIIA, EIIB and finally to the substrate as it is transported across the membrane. The organization of the *C. glutamicum* PTS for fructose, glucose and sucrose are shown in Fig. 1. *ptsG *(cg1537) encodes the glucose‐specific membrane integral EIIBCA transporter, while *ptsS* (cg2925) encodes the sucrose‐specific EIIBCA, and *ptsF* (cg2120) the fructose‐specific EIIABC (Fig. 1). A fourth putative PTS belongs to the L‐ascorbate‐type family and is encoded by *rmpC* (cg3365) (not depicted in the graphic), but no function has been assigned to this system so far (Uhde *et al.*, 2013).

It has been proposed that the PTS can be described as a central ‘cellular functioning unit’ (CFU), regulating the cells quest for food (Lengeler, 2000; Lengeler and Jahreis, 2009; Lengeler, 2015). However, to fully describe CFUs, precise knowledge about spatio‐temporal localization of its components is required. There is surprisingly little knowledge on subcellular localization of PTS components. Early immune‐gold labeling with the *E. coli* EII^mtl^ showed membrane localization that can be interpreted as patchy with no preferred subcellular enrichment (Maddock and Shapiro, 1993). However, deconvolved images and plasmid born expression do not allow for unambiguous differentiation between a uniform and a patchy localization. The general components of the *E. coli* PTS, EI and HPr in contrast localize to the cell poles (Lopian *et al.*, 2010; Govindarajan *et al.*, 2013), and HPr localization has been shown to change in presence of the transport substrate from polar to dispersed throughout the cytoplasm (Lopian *et al.*, 2010). In contrast, EI and HPr of *B. subtilis* have been shown to be dispersed throughout the cytoplasm (Rothe *et al.*, 2013).

Here, we describe the localization of two specific EII components and the general PTS components HPr and EI in *C. glutamicum*. We have constructed translational fusions of *ptsG*, *ptsF*, *ptsH* and *ptsI* encoding PtsG (EII^glc^), PtsF (EII^fru^), HPr and EI with different fluorescent proteins. PtsG, PtsF and HPr constructs were designed as allelic replacements, ensuring native genetic control, whereas EI construct is plasmid‐based in a *ptsI* deficient strain, and all constructs were shown to be fully functional. Widefield fluorescence microscopy revealed that the general components HPr and EI are diffused in the cytoplasm, in contrast to EII complexes, that localize as dynamic clusters in the cell membrane. PtsG and PtsF EII components exclude each other within the membrane compartment, but PtsF co‐localize with components of the respiratory chain, ruling out a specific membrane domain for carbohydrate transport only. We observed an increase in PTS EII cluster size when the specific sugar substrate was present. This increase in cluster size coincided with a decrease in cluster number. Using photo‐activated single molecule fluorescence microscopy (PALM) we were able to quantitatively address PTS dynamics. PALM data clearly show that PTS EII cluster are covering a larger membrane area when their transport substrate is present. Importantly, under these conditions the complexes do not contain more EII molecules, but rather reduce protein density within clusters. Thus, actively transporting PTS permeases are spreading apart and non‐transporting complexes are densely packed. This dynamic arrangement of the PTS offers a simple mechanism for efficient membrane occupancy under varying nutrient conditions.

## Results

### Construction of functional PTS fusion proteins

**Figure 2:**
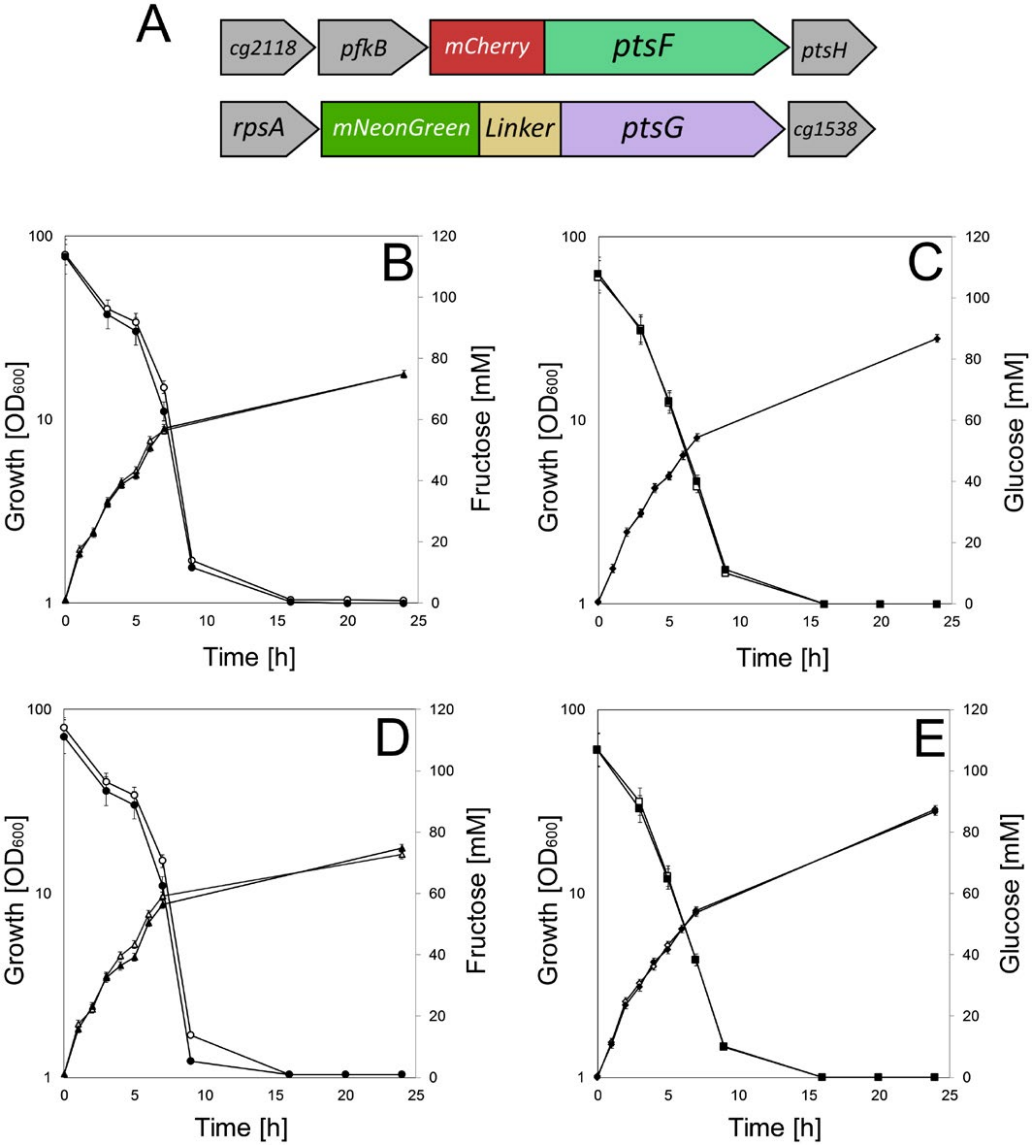
Fluorescent fusions of PtsF and PtsG are fully functional**.** (A) Genetic arrangement of fluorescent fusions constructed via allelic replacement of *ptsF::mCherry‐ptsF* and *ptsG::mNeonGreen‐linker‐ptsG*. Growth and sugar consumption of *C. glutamicum* strains CGM001 (B), CGM002 (C) and CGM003 (D, E) (filled symbols) versus wild type RES 167 (open symbols) on CGXII containing (B, D) 100 mM fructose and (C, E) 100 mM glucose. Fructose consumption (circles), glucose consumption (squares), growth on fructose (triangles), and growth on glucose (diamonds) are indicated. Each point represents biological triplicates and standard deviation is indicated.

To investigate subcellular localization and membrane occupancy of PTS EII permeases, we constructed *C. glutamicum* strains with fluorescent fusions of PtsF and PtsG, resulting in strains CGM001 and CGM002 expressing mCherry‐PtsF and mNeonGreen‐PtsG respectively (Table S2). To investigate a possible influence of the general PTS components in the dynamics of PtsF/G, we constructed the strains CGM007 and CGM009 expressing HPr‐mVenus and eCFP‐EI respectively (Table S1). Despite multiple attempts, the construction of an allelic replacement fluorescent fusion of EI was not possible, therefore we utilized a fully functional plasmid‐based eCFP‐EI. We have not included the sucrose specific PTS, since sucrose is a disaccharide composed of fructose and glucose and we wanted to first test for the specific influence of glucose and fructose. *C. glutamicum* imports and phosphorylates sucrose via the sucrose‐specific PtsS. The phosphorylated sucrose is cleaved intracellularly and the resulting fructose molecule is exported and taken up by the fructose specific PtsF. Hence, a clean separation between sucrose and fructose effects is not easily possible. To check for potential co‐localization of the glucose and fructose specific EII permeases a double labeled strain was constructed. To this end, CGM001 was used as a background for incorporation of *mNeonGreen‐linker‐ptsG* via allelic replacement, resulting in the double‐labeled strain *ptsF::mCherry‐ptsF*, ptsG::*mNeonGreen‐linker‐ptsG* (CGM003) (Fig. 2A). In order to gain quantitative single molecule resolution data of PTS localization and clustering patterns, strains containing PtsF (CGM004) and PtsG (CGM005) tagged with photoactivatable mCherry (PAmCherry) were constructed for PALM microscopy. To avoid overexpression and artificial expression heterogeneity, the fusion constructs were all inserted as allelic replacement in the genome of wild type *C. glutamicum* (RES 167) cells. To test the functionality of constructed fusion proteins, growth experiments and sugar consumption assays via HPLC were performed. Strains CGM001, expressing mCherry‐PtsF (Fig. 2B), CGM002 expressing mNeonGreen‐PtsG (Fig. 2C) and the double labeled strain CGM003 displayed wild type like growth behavior (Fig. 2D and E). The growth rates of wild type did not differ from the constructed strains and ranged from 0.28 to 0.29 h^−1^ (Table 1), showing that the tagged PTS were functioning wild type‐like. We also measured sugar consumption rates in CGXII supplemented with 2% fructose or 2% glucose. The specific sugar consumption rates q_s_ were highly similar for wild type and mutant strains (Table 1). The measured q_s _values for the wild type of around 0.23 g g^−1^ h^−1^ for glucose and 0.14 g g^−1^ h^−1^ for fructose are very well within the expected range for the observed growth rate (Kawaguchi *et al.*, 2018). The strain carrying the fluorescently labeled PtsG had consumption rates of 0.23 g g^−1^ h^−1^ for glucose and the strain with the PtsF fusion construct displayed consumption rates of 0.17 g g^−1^ h^−1^ for fructose. In summary, we conclude that the constructed fusions are fully functional. It should be noted here, that in a process to gain functional translational fusions, several constructs were made that were all not further analyzed when they turned out to be non‐functional.

**Table 1:** Growth rates and glucose consumption rates. Growth rate [h^−1^] and substrate uptake [g g^−1^ h^−1^] of RES 167 WT and strains CGM001, CGM002, CGM003 and CGM005 in CGXII supplemented with glucose and fructose.

	Glucose		Fructose	
	Growth rate [h^−1^]	Uptake (qs) [g g^−1^ h^−1^]	Growth rate [h^−1^]	Uptake (qs) [g g^−1^ h^−1^]
WT	0.285	0.23 ± 0.003	0.289	0.14 ± 0.005
CGM001	–	–	0.286	0.17 ± 0.004
CGM002	0.280	0.23 ± 0.001	–	–
CGM003	0.290	0.23 ± 0.001	0.280	0.17 ± 0.004
CGM005	0.285	0.23 ± 0.001	–	–

Protein localization studies using translational fusions can be hampered by protein degradation and subsequent imaging of free fluorophore. Therefore, we investigated protein degradation by western blotting with anti‐mCherry for mCherry and PAmCherry fusions, and in‐gel fluorescence for mNeonGreen, eCFP and mVenus fusions. For each strain, it was possible to identify the one band corresponding to their full length fusion protein and when present, oligomers, revealing that no major degradation was present (Fig. S1). We therefore conclude that localization studies with these strains should reveal the native localization of the full length PTS EII permeases.

### Localization of the general PTS components HPr and EI

**Figure 3:**
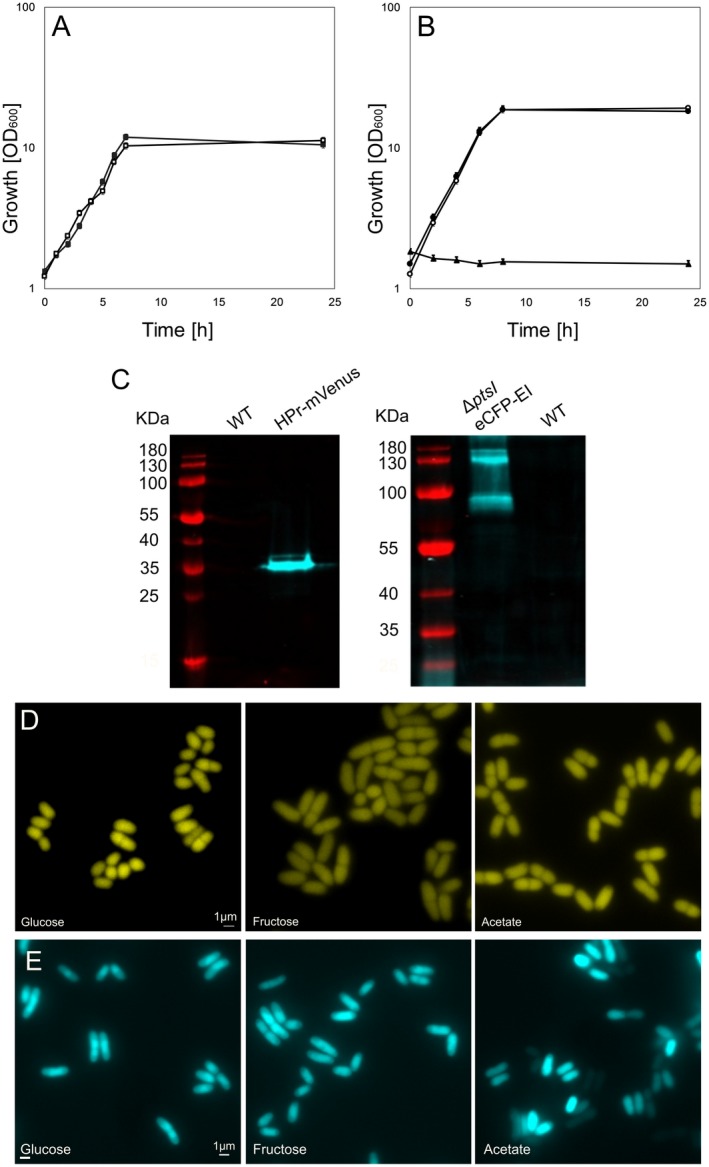
HPr and EI localize dispersely in the cytoplasm. A. Growth of *C. glutamicum* strain CGM007 (*hpr::hpr‐mVenus*) (filled squares) versus wild type RES 167 (open squares) in CGXII containing 100 mM glucose. B. Growth of *C. glutamicum* strain CGM009 (*ΔptsI *with pEKEx2_eCFP‐EI) (filled circles) versus wild type RES 167 (open circles) and CGM008 (*ΔptsI*) (filled triangles) in CGXII containing 100 mM glucose. C. In‐gel fluorescence of crude extract of RES 167, CGM007 and CGM009. The expected size of the HPr‐mVenus fusion protein is 35,5 kDa and eCFP‐EI is 85,61 kDa. D. Epifluorescence microscopy images of *C. glutamicum* expressing HPr‐mVenus grown for 5 h in indicated carbon sources. E. Epifluorescence microscopy images of *C. glutamicum* expressing eCFP‐EI grown for 5 h in indicated carbon sources.

Previous work in *E. coli* has shown that the general components EI and HPr might be localized in a substrate dependant manner (Lopian *et al.*, 2010). Therefore, we wanted to address the localization of the general PTS components in *C. glutamicum*. Similar to the strategy employed for the EII components, we wanted to construct functional allelic replacements of EI and HPr. We succeeded in the generation of a HPr‐mVenus fusion (h*pr::hpr‐mVenus,* CGM007), and despite several attempts, we were not able to construct an allelic replacement‐based EI fusion, therefore we utilized a plasmid‐based eCFP‐EI fusion in a *ΔptsI* background (CGM009). The strains had wild‐type growth in CGXII with glucose as sole carbon source (Fig. 3A and B), confirming the functionality of HPr and EI. In‐gel fluorescence revealed the expected size of HPr fused to mVenus, as well as monomers, dimers and trimers of EI, with no signs of protein degradation (Fig. 3C). Widefield microscopy of cells grown in CGXII for 5 h with different carbon sources revealed that both HPr and EI localize dispersly in the cytoplasm regardless of the presence or absence of PTS sugars. We noticed that EI seems to localize more densely over the nucleoid when cells were grown in glucose or fructose (Fig. 3D and E). The corrected total fluorescence (CTF) was calculated with ImageJ based on the obtained fluorescence microscopy images and corrected for the cell area, and the values for cells expressing eCFP‐EI and HPr‐mVenus grown in different carbon sources are: glucose EI = 5665.92 ± 2212.942, HPr = 2117.72 ± 619.75, fructose EI = 5152.70 ± 1600.91, HPr = 1940.18 ± 440.30 and sodium acetate EI = 4983.00 ± 1918.99, HPr = 1807.13 ± 311.95. This suggests an inverse correlation between cell size and CTF readings, as cells grown in glucose (1.64 ± 0.42 µm^2^) tend to be smaller than cells grown in fructose (1.91 ± 0.36 µm^2^) and acetate (2.26 ± 0.72 µm^2^). HPr and EI expressions are therefore constitutive. Overall, our data with eCFP‐EI and HPr‐mVenus show that in *C. glutamicum*, the general PTS components EI and HPr do not form clusters.

### *C. glutamicum* EII^fru^ EII^glc^ spatial dynamics upon presence or absence of the transported sugars

**Figure 4:**
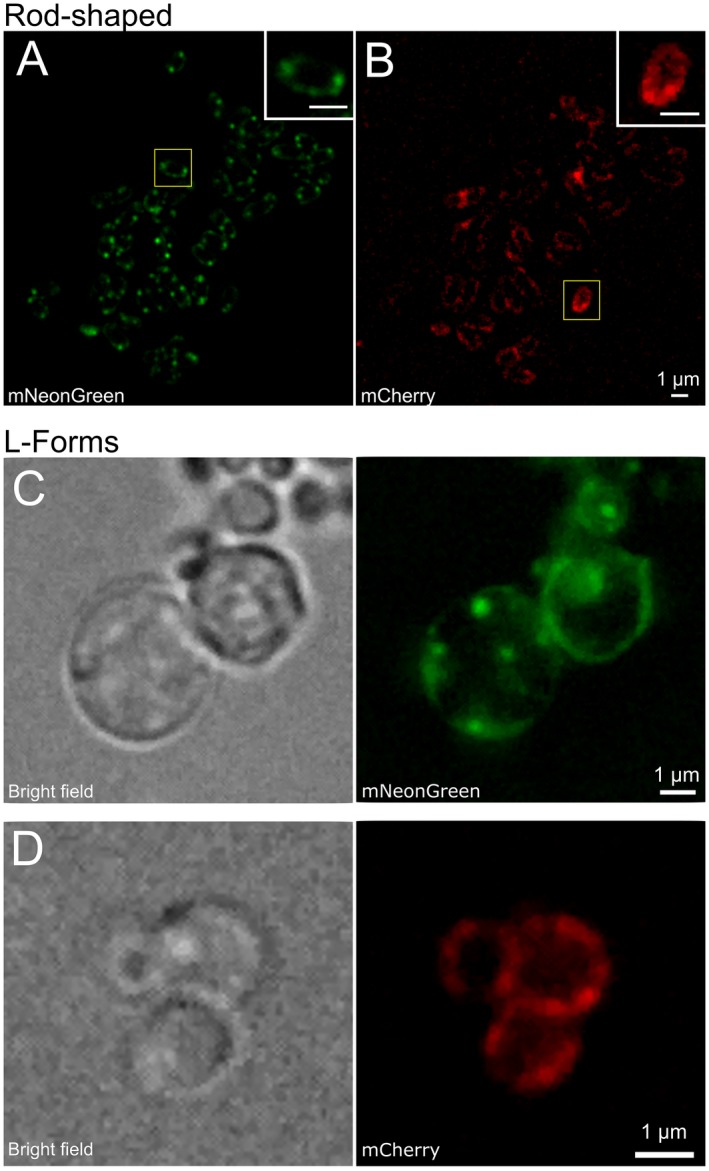
PtsF and PtsG localize as clusters distributed along the cytoplasmic membrane. Epifluorescence microscopy images of *C. glutamicum* expressing mCherry‐PtsF and mNeonGreen‐PtsG under different growth conditions: Rod‐shaped cells grown in CGXII with (A) glucose and (B) fructose as sole carbon sources. L‐Form cells grown in MSM/CGXII with (C) glucose and (D) fructose as sole carbon sources in presence of 200 µg/ml of DCS.

Next wanted to investigate whether PTS EII proteins distribute uniformly or in clusters along the cytoplasmic membrane under aerobic conditions in presence of the transported sugars. To this end, fluorescence microscopy was performed on *C. glutamicum* strains expressing mNeonGreen‐PtsG and mCherry‐PtsF grown in CGXII supplemented with the transported sugars as sole carbon sources. Analysis of fluorescence images showed that both proteins form membrane embedded clusters that localize punctually within the cell membrane with no preferred positions (Fig. 4A and B). EII^fru^ and EII^glc ^foci of varying intensity were observed, suggesting complexes with different amounts of proteins. Most cells contained few bright and intense foci that were randomly distributed. However, bright foci seemed to be present with higher frequency close to cell poles (Fig. 4A and B).

We next wanted to test whether cell shape and the surface/volume ratio might have an influence on the PTS EII permeases. Therefore, we analyzed PTS localization in L‐form bacteria, generated out of the corresponding strains. Cells expressing mNeonGreen‐PtsG and mCherry‐PtsF were grown in MSM/CGXII medium supplemented with the transported sugars in presence of D‐cycloserine (DCS). DCS is a cyclic analogue of D‐alanine, acting against alanine racemase (Alr) and D‐alanine:D‐alanine ligase (Ddl), two crucial enzymes in the cytosolic stages of peptidoglycan synthesis, bypassing the need to block cell wall synthesis genetically. As DCS was previously used in *Mycobacterium tuberculosis* (Prosser and de Carvalho, 2013), which shares the characteristic cell wall common to all *Corynebacterineae*, it was chosen for L‐form formation with *C. glutamicum*. L‐form bacteria require an osmotically stabilized medium. Usually, the osmoprotective environment would be achieved by adding sucrose to the media, but since this sugar is taken up by the sucrose specific PtsS, we complemented the L‐form medium with xylose, a carbohydrate that does not support growth of *C. glutamicum* (Fig. S2) (Kawaguchi *et al.*, 2006). Fluorescence microscopy with L‐forms revealed the same PtsF and PtsG clustering that was observed in rod‐shaped cells (Fig. 4C and D). We conclude from this, that cluster formation of PTS EII permeases may be an intrinsic property of these enzymes and not dependent only on cell shape or surface/volume ratios.

**Figure 5:**
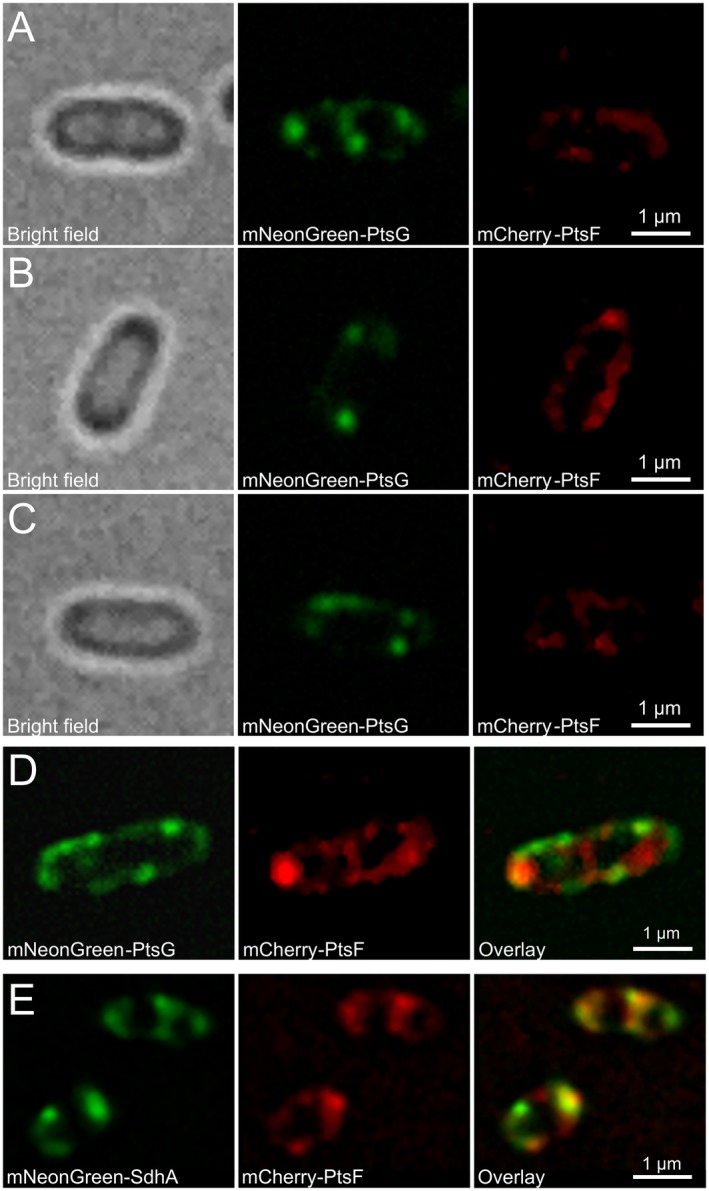
PtsF and PtsG clustering changes upon presence or absence of transported substrate. Epifluorescence microscopy images of *C. glutamicum* expressing mCherry‐PtsF and mNeonGreen‐PtsG under different growth conditions. Cells grown in CGXII with 2% (A) glucose, (B) fructose or (C) acetate as sole carbon sources. (D) mNeonGreen‐PtsG and mCherry‐PtsF in CGXII with 2% glucose and fructose. (E) mNeonGreen‐SdhA and mCherry‐PtsF in CGXII with 2% fructose.

We next wanted to investigate whether presence or absence of the specific PTS carbohydrates influence the PTS EII permeases localization and expression. To this end, the strains containing fluorescent reporters linked to the EII complexes were grown in CGXII minimal medium with 2% glucose, fructose or acetate as sole carbon sources to early exponential phase, and fluorescent microscopy was subsequently performed. Acetate is taken up by diffusion and/or via MctC, a secondary transporter that belongs to the class of sodium solute symporters (Jolkver *et al.*, 2009). Importantly, both proteins are expressed in the absence of their specific transported sugars (Fig. 5A–C), and no differences among early, mid and late log phases were observed (data not shown). We also observed EII clustering when cells were grown in acetate (Fig. 5C). Observed PtsG/F foci did not show evident co‐localization (Fig. 5D), suggesting that phosphotransferase systems localize independently within the membrane. This is an important observation, because the general PTS components EI and HPr are required for activation of both EII complexes. As a control we analyzed localization of the EII^fru^ complexes with a protein from the respiratory chain. Here, we have chosen the succinate dehydrogenase subunit A (SdhA) in combination with PTS EII^Fru^. We did observe a large degree of co‐localization between PtsF and SdhA (Fig. 5E). On a scale that can be analyzed in diffraction limited microscopy, our data rule out that in *C. glutamicum,* components of the respiratory chain and the PTS would always occupy specific and different membrane domains.

**Figure 6:**
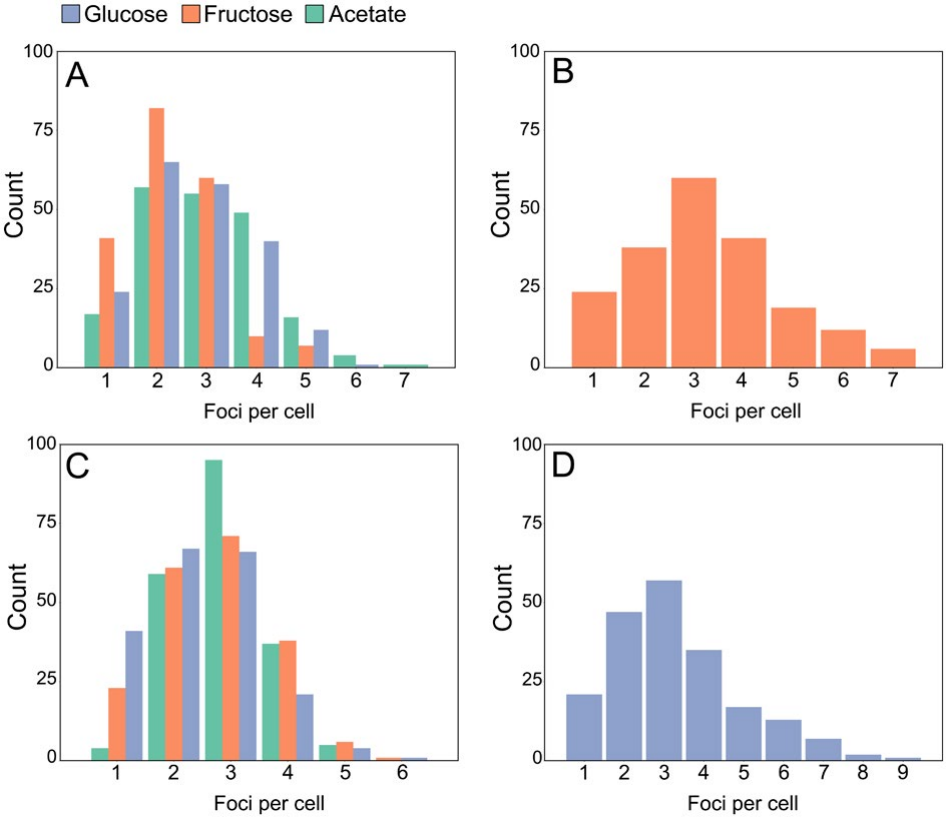
mCherry‐PtsF and mNeonGreen‐PtsG foci number decreases in presence of transported sugar. Number of foci per cell of (A) mCherry‐PtsF in rod shaped cells, (B) mCherry‐PtsF in L‐form cells. (C) mNeonGreen‐PtsG in rod shaped cells, (D) mNeonGreen‐PtsG in L‐form cells.

After the observation that clustering and expression of PTS EII occurs even in absence of the transported substrate, we next wanted to determine the influence of different carbon sources on PTS foci number per cell, foci area, fluorescence, and how much of the membrane space is covered by PTS. Analysis of fluorescent images of *ptsF::mCherry‐ptsF*, *ptsG::mNeonGreen‐Linker‐ptsG* both in rod shaped cells and L‐forms grown in minimal medium in presence of different carbon sources revealed that the number of PtsF/G clusters per cell was significantly decreased in presence of the transported sugar in rod‐shaped cells (Fig. 6 and Table 2). Statistically, the number of PtsF clusters per cell was equal in acetate and glucose treatments, and differed in rod‐shaped and L‐form cells grown in fructose. Regarding PtsG foci number per cell in rod‐shaped, cells grown in fructose or acetate did not differ statistically, while when grown in glucose, the values were significantly lower. L‐form cells showed a tendency to have more PTS foci, having higher averages (3.3 per cell for both PtsF/G), being statistically different from all the other treatments. The absence of a cell wall in L‐forms results in significantly larger cells (mean cell area values: rod‐shaped 3.6 µm^2^, L‐forms 8.9 µm^2^), which leads to a drastic reduction in the cell surface/volume (S/V) ratio. Despite being grown in medium containing the same carbon source, the observed increase in number of PtsF/G clusters in L‐forms suggests that PTS EII foci pattern can be changed in response to variations in cell area, volume or morphology, although these changes are proportional to the larger cell size of L‐forms.

**Table 2:** Number of PTS EII foci per cell in various growth conditions. Mean values of foci number per cell of mCherry‐PtsF and mNeonGreen‐PtsG. ‘Fructose’, ‘Glucose’ and ‘Acetate’ represent measurements of rod‐shaped cells in CGXII medium with the respective carbon sources. ‘L‐Forms’ represent measurements of L‐Form cells in MSM/CGXII supplemented with the transported substrate. Significant statistical differences according to multiple comparison tests after Kruskal–Wallis are represented as letters next to each value.

	Fructose	Glucose	Acetate	L‐Forms
PtsF	2.4 (b)	2.6 (a)	2.9 (a)	3.3 (c)
PtsG	2.8 (a)	2.5 (b)	2.8 (a)	3.3 (c)

**Figure 7:**
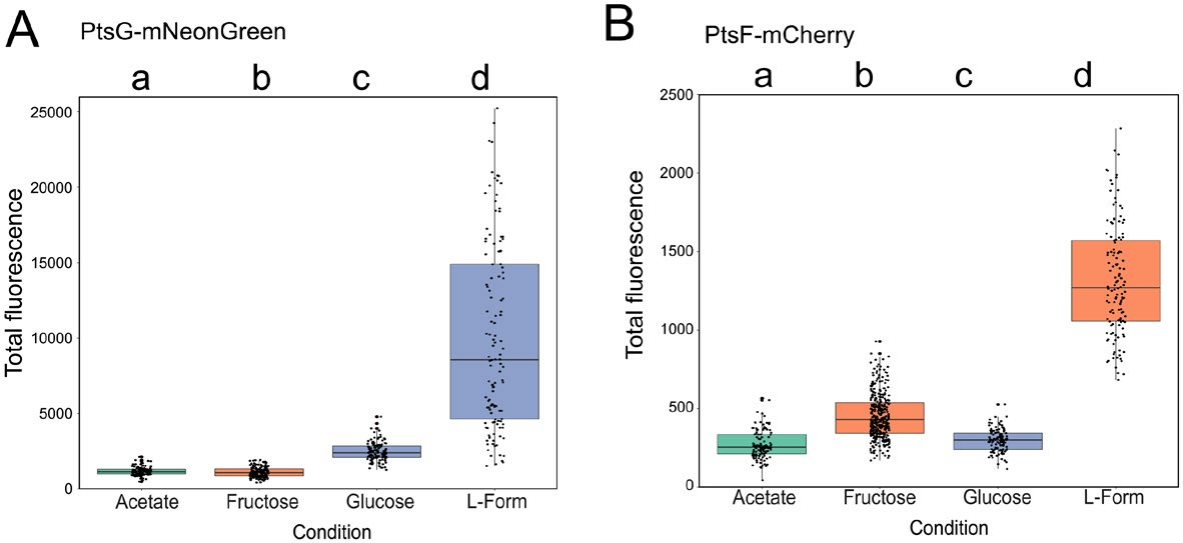
*C. glutamicum* PTS expression increases in presence of transported sugar. Corrected total fluorescence of (A) mNeonGreen‐PtsG and (B) mCherry‐PtsF. ‘Fructose’, ‘Glucose’ and ‘Acetate’ represent rod‐shaped cells in CGXII medium with the respective carbon sources. ‘L‐form’ represents L‐form cells in MSM/CGXII supplemented with fructose for PtsF and glucose for PtsG. Significant statistical differences according to multiple comparison tests after Kruskal–Wallis are represented as letters above each graph.

Previous studies have revealed that the expression of the *C. glutamicum* PTS is induced when cells are cultured in presence of the transported sugar (Parche *et al.*, 2001; Engels and Wendisch, 2007; Engels *et al.*, 2008; Tanaka *et al.*, 2008). In order to test the influence of different carbon sources on PTS foci, PtsF/G Corrected Total Fluorescence (CTF) was calculated with ImageJ based on the obtained fluorescence microscopy images and corrected for the cell area (Fig. 7A and B). Analysis of CTF of tagged proteins served as a proxy to assess levels of protein expression and concentration, and both PtsF and PtsG were similar regarding the fluorescence readings in this work. Although all the treatments were statistically different from each other, CTF of both proteins increased in presence of the transported substrate and decreased in its absence. This indicates that indeed, *ptsF* and *ptsG* expression was induced in presence of the transported substrate.

**Figure 8:**
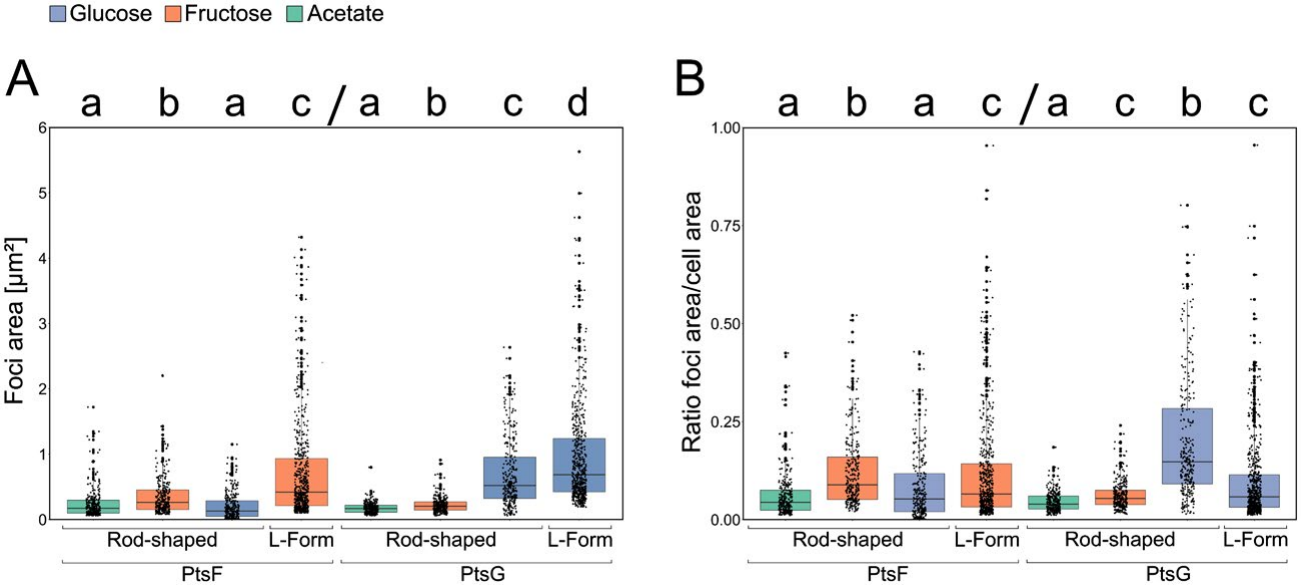
mCherry‐PtsF and mNeonGreen‐PtsG foci area increases upon presence of transported sugar. A. mCherry‐PtsF and mNeonGreen‐PtsG fluorescent foci area in rod‐shaped and L‐Form cells in CGXII supplemented with different carbon sources. B. Ratio foci area and cell area under different carbon sources and cell shapes. ‘L‐Form’ represent L‐Form cells in MSM/CGXII supplemented with the transported sugar. Significant statistical differences according to multiple comparison tests after Kruskal–Wallis are represented as letters above each graph. Different letters indicate differences within the same PTS protein.

The areas of the individual PtsF/G foci of cells grown in different carbon sources are summarized in Fig. 8A and Table 3. In rod‐shaped cells, PtsF foci area values were statistically similar when cells were grown in glucose or acetate, and significantly higher in fructose. Likewise, PtsG foci area in glucose was three times higher than in fructose, and four times than in acetate. However, unlike PtsF, the PtsG cluster area values obtained for cells grown in fructose were higher than cells grown in acetate. L‐Form cells exhibited the highest PtsF/G foci areas compared to every other condition in rod‐shaped cells, suggesting that a larger membrane area create more space to be occupied by transmembrane proteins.

**Table 3:** PtsF and PtsG cover different membrane surface areas. Mean values of foci area [µm^2^] of mCherry‐PtsF and mNeonGreen‐PtsG. ‘Fructose’, ‘Glucose’ and ‘Acetate’ represent measurements of rod‐shaped cells in CGXII medium with the indicated carbon sources. ‘L‐Forms’ represent measurements of L‐Form cells in MSM/CGXII supplemented with the transported substrate (fructose or glucose). Values are in µm^2^. Significant statistical differences according to Multiple comparison tests after Kruskal–Wallis are represented as letters next to each value.

	Fructose	Glucose	Acetate	L‐Forms
PtsF	0.35 (b)	0.18 (a)	0.24 (a)	0.71 (c)
PtsG	0.22 (a)	0.70 (b)	0.17 (c)	1.00 (d)

In order to know how much of the cytoplasmic membrane is occupied by PTS, the ratio foci area/cell area (Fig. 8B) was calculated. Rod‐shaped cells had the highest ratios when in presence of the transported sugar, meaning that more membrane space is reallocated for PTS proteins in such conditions. In general, L‐forms exhibited larger PTS clusters, in a higher overall number, and fluorescence. This leads to the logical assumption that a larger cell area increases the area available for protein insertion, possibly resulting in more transmembrane PTS proteins. However, the foci area/cell area ratio of L‐Forms was not higher than other rod‐shaped cells. In fact, despite PtsF L‐form values being higher than in rod‐shaped cells grown in glucose or acetate, they were lower than rod‐shaped cells in fructose (Table 4). Roughly, the same was observed for PtsG: even though L‐forms had a higher foci area/cell area ratio than rod‐shaped cells in general, there was no statistical difference between their values and rod‐shaped cells grown in fructose, a condition where PtsG is hardly induced.

**Table 4:** PTS EII complex surface coverage. Foci area/cell area ratio of mCherry‐PtsF and mNeonGreen‐PtsG. ‘Fructose’, ‘Glucose’ and ‘Acetate’ represent measurements of rod‐shaped cells in CGXII medium with different carbon sources. ‘L‐Forms’ represent measurements of L‐Form cells in MSM/CGXII supplemented with the transported substrate. Significant statistical differences according to multiple comparison tests after Kruskal–Wallis are represented as letters next to each value.

	Fructose	Glucose	Acetate	L‐Forms
PtsF	0.10 (b)	0.05 (a)	0.06 (a)	0.08 (c)
PtsG	0.06 (c)	0.20 (b)	0.04 (a)	0.11 (c)

### Single molecule localization microscopy reveals spatial rearrangement of EII^glc^ in presence of glucose

**Figure 9:**
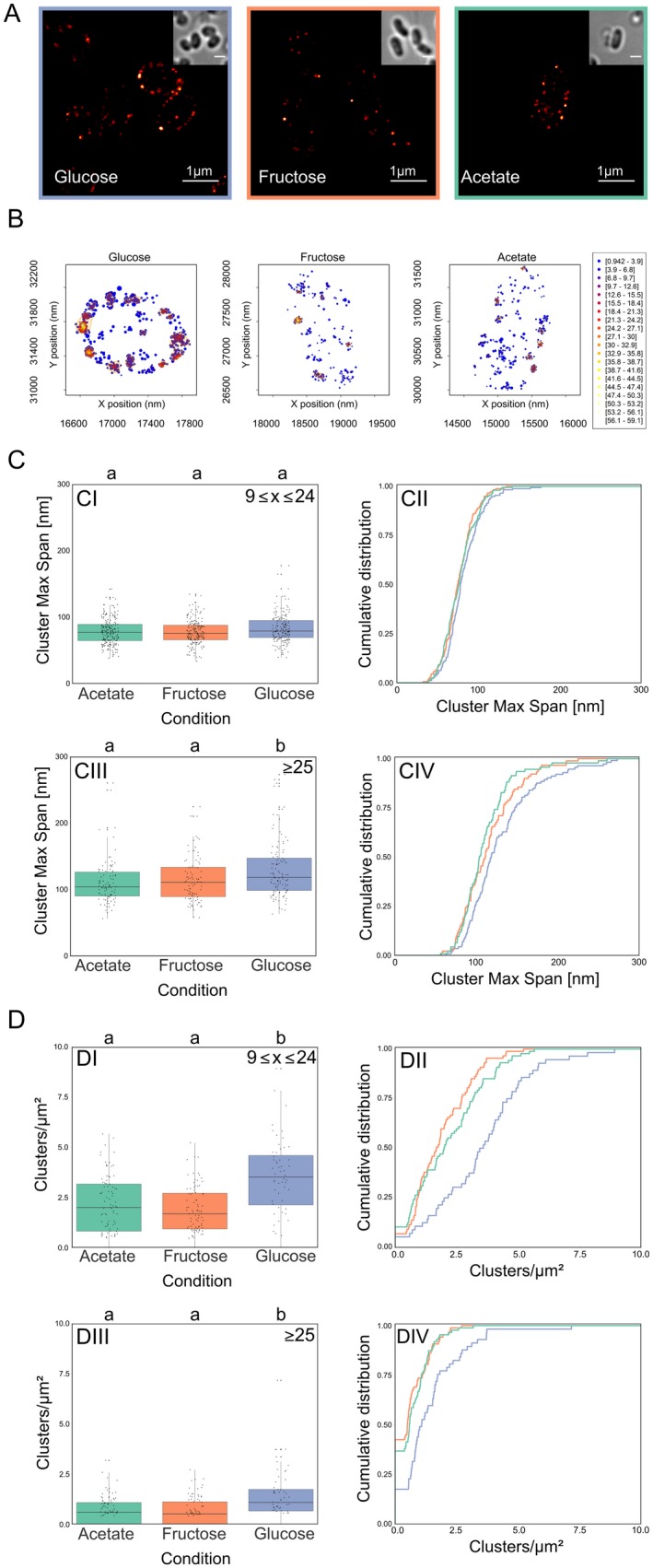
Single molecule localization microscopy reveals dynamic PAmCherry‐PtsG clustering. A. Super resolution images of *C. glutamicum* strain CGM005 expressing PAmCherry‐PtsG under different growth conditions. Cells were grown in CGXII with indicated carbon source. Insets show transmitted light images to see cell outlines. Scale bar is 1 µm. B. Plot of detected single molecule localization events localization and clusters identification for a representative cell for each tested condition. Localizations are color coded according to the local event density while the plotted radius is indicating localization precision. The identified clusters are highlighted by orange contours. C. PtsG clusters maximum span increase in presence of glucose. D. PtsG clusters are more abundant in presence of glucose. Each parameter is analyzed via (CI, CIII, DI and DIII) boxplot and (CII, CIV, DII and DIV) cumulative distribution function. Clusters composed of (CI, CII, DI and DII) 9 ≤ x ≤ 24 events, and (CIII, CIV, DIII and DIV) >25 events were analyzed. Significant statistical differences according to multiple comparison tests after Kruskal–Wallis are represented as letters above each graph. Different letters indicate statistical differences within the same PTS protein.

The data obtained with widefield microscopy suggested that PTS EII cluster rearrange when their specific transport substrate is present. However, epifluorescence microscopy is limited by the diffraction limit, and a series of fundamental information about PTS EII clusters, such as density or number of molecules cannot be obtained. Therefore, the next step we took toward a deeper and more quantitative understanding of PTS EII complex dynamics was to use single molecule photo‐activated localization microscopy (PALM) data. To this end, we constructed strains tagged with photoactivatable mCherry (PAmCherry) *ptsG::PAmCherry‐Linker‐ptsG* and *ptsF::PAmCherry‐ptsF* and showed that they are fully functional as judged by sugar uptake and growth rates (Fig. S3). The observed clustering pattern in epifluorescence was confirmed by PALM data of cells expressing PAmCherry‐PtsG (strain CGM005) (Fig. 9 A and B). Also, PAmCherry‐PtsF formed membrane embedded clusters, but the low number of events detected per cell made it impossible to obtain significant statistical analysis that would show cluster density changes (Fig. S4A–E). However, we observed a tendency that is in accord with the data obtained for PtsG.

For analysis of PALM data, we first needed to precisely define what a valid cluster is. Clusters are defined as regions of high density separated by regions of lower density, and the distribution of our data suggests that there are three populations separated by the amount of fluorescence events composing each cluster: (i) *X* < 10, (ii) 10 ≤ *X* ≤ 24, and (iii) *X* ≥ 25 events (Fig. S5). The first population could in theory still be composed of PTS complexes close to each other by mere coincidence. Although this randomness effect is never fully absent, it decreases with increasing cluster size. Therefore, clusters composed of ≥10 and ≥25 events were analyzed regarding their maximum span, density, number of events per µm^2^, and number of clusters per µm^2^. The stretched exponential distribution of PTS cluster size is reminiscent to that observed with chemotaxis receptors (Greenfield *et al.*, 2009) and therefore suggests a stochastic self‐assembly process. We detected around 177.65 ± 92.48 PtsG events per µm^2^ cell area in glucose, 111.22 ± 49.51 events per µm^2^ in fructose, and 114.69 ± 60.28 events per µm^2^ in acetate.

**Figure 10:**
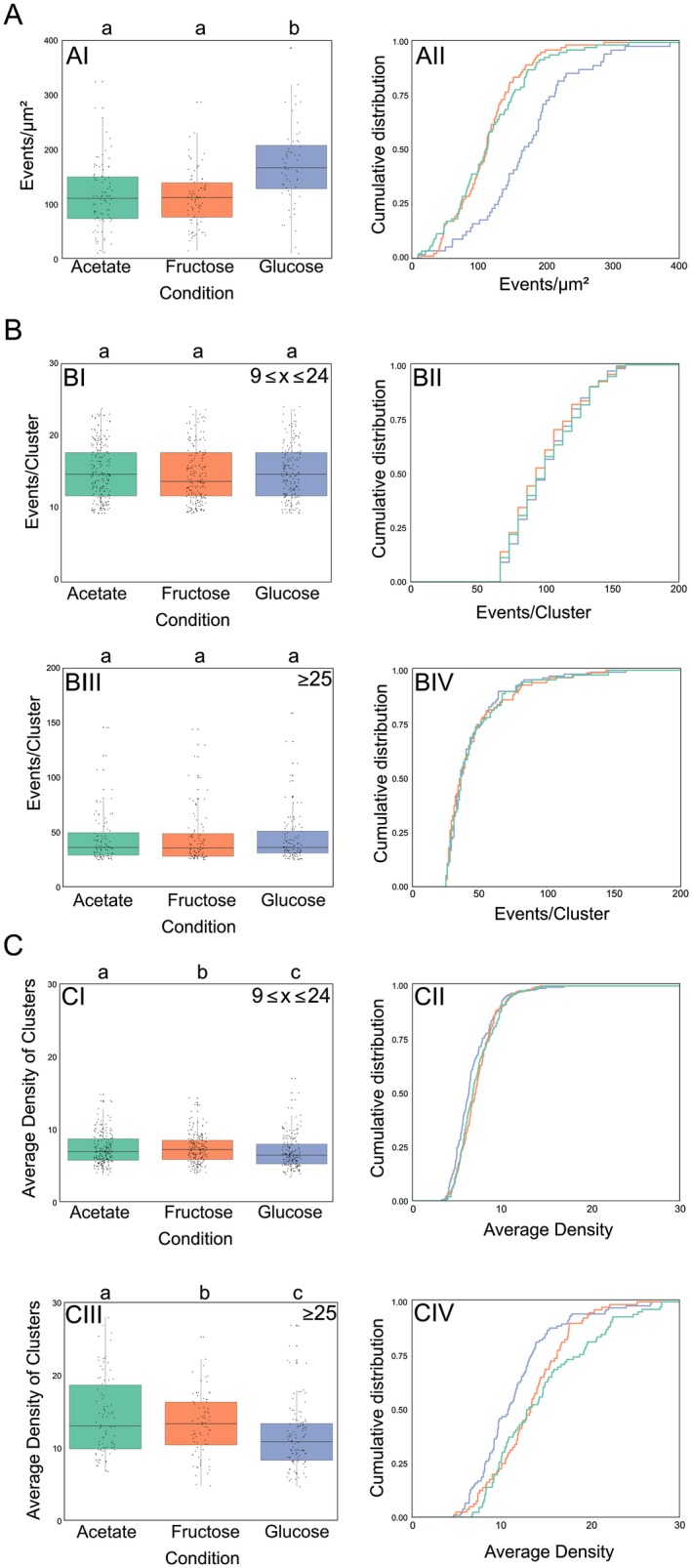
PTS cluster decrease protein density in presence of the transport substrate. Super resolution PALM data of CGM005 strain (*ptsG::PAmCherry‐ptsG*) grown in CGXII supplemented with 2% of the indicated carbon sources. A. PtsG number of events per µm^2^ increases in presence of glucose. B. PtsG clusters are composed by the same amount of molecules regardless of the carbon source. Plots represent events per cluster in clusters composed of >25 molecules. C. PtsG clusters have lower average density in presence of glucose. Each parameter is analyzed via (AI, BI, BIII, CI and CIII) boxplot and (AII, BII, BIV, CII and CIV) cumulative distribution function. Clusters composed of (BI, BII, CI and CII) 9 ≤ *x* ≤ 24 events, and (BIII, BIV, CIII and CIV) >25 events were analyzed. Significant statistical differences according to multiple comparison tests after Kruskal–Wallis are represented as letters above each graph. Different letters indicate statistical differences within the same PTS protein.

The increase in foci area observed in epifluorescence brought up the question whether the cluster area increases due to an increase in number of PTS complexes present in each cluster, or to a rearrangement of the same number of EII permeases per cluster. At single molecule resolution, foci area can be estimated by the cluster maximum span (Fig. 9C), which is the maximum distance between two events belonging to the same cluster. PAmCherry‐PtsG clusters from population C exhibited significantly higher max span in the presence of glucose when compared to fructose or acetate, which exhibited no statistical difference among each other, corroborating our findings with epifluorescence, where in presence of the transported sugars, PtsG/F covered a larger membrane area. The analysis of these same populations of clusters revealed that cells in presence of glucose exhibited significantly higher number of PtsG clusters per µm^2^ when compared to fructose and acetate, which exhibited no differences among each other (Fig. 9D). CTF readings of epifluorescence images showed increased values when cells expressing mCherry‐PtsF and mNeonGreen‐PtsG were grown in presence of the transported sugars, suggesting increased expression of PTS complexes under these conditions. This data correlates with our PALM data for PamCherry‐PtsG (Fig. 10A). In presence of glucose, a significantly higher number of events per µm^2^ was observed, meaning that the number of PtsG in the cytoplasmic membrane increases with the addition of glucose in the medium, again confirming induction of *ptsG* in presence of glucose as expected. Our epifluorescence data suggested that in presence of the transported sugar, PtsF/G assemble in larger complexes, while the overall number of clusters per cell decreases. However, our PALM analysis of cells expressing PAmCherry‐PtsG in glucose showed an increase in the number of clusters and events per µm^2^. Single molecule detection allowed visualization of clusters with fewer proteins, thereby explaining the apparent discrepancy to the widefield microscopy data. While in widefield the number of bright, visible foci decreased (while their area increased) when the transport substrate was present, in PALM we observed more clusters with lower protein numbers that likely account from substrate induced gene expression.

Although the CTF of cells increases in presence of glucose, PALM data showed that the number of PAmCherry‐PtsG events per cluster remain the same independently of the carbon source, suggesting that the carbon source does not affect the number of PTS proteins present in each cluster (Fig. 10B). This is an important finding since larger cluster size could have been the trivial consequence of more protein in the cell. Since we have found that the number of PTS EII molecules in a cluster remained similar in presence or absence of the transported substrate, we wanted to analyze the protein density in individual clusters. The local density is defined as the number of events present in a squared area of side 50 nm centered on the event. The average density of events is defined as the arithmetic average of local density of the events composing the cluster. PAmCherry‐PtsG clusters of population C exhibited significant lower average density values in presence of glucose (Fig. 10C), meaning that PtsG complexes belonging to the same cluster localize further apart from each other when in presence of glucose, thereby occupying a larger membrane area.

## Discussion

The transport of sugars via PTS allows the highly effective transport even under low external carbohydrate concentrations. Yet, a second, similarly important feature of PTS transport is that the PTS is composed of a sensory part and a regulatory part (Lengeler, 2000). The general PTS component HPr is involved in regulation and e.g. phosphorylates the transcriptional regulator BglG in *E. coli* (Gorke and Rak, 1999) and and LciT in *Bacillus subtilis* (Lindner *et al.*, 1999). For HPr, a polar localization that is alleviated when transport substrates are present was described in *E. coli* (Lopian *et al.*, 2010). These data were in line with early suggestions that the PTS complex should act in multi‐protein complexes (Rohwer *et al.*, 1998), thereby improving its function (Norris *et al.*, 1999). Our data shows that in *C. glutamicum*, the general components HPr and EI are diffused in the cytoplasm regardless of the presence or absence of PTS sugars. This is in line with previous works that show a constitutive expression of HPr and EI in other organisms (Stulke *et al.*, 1997; Rothe *et al.*, 2013). Based on our results, we do not expect that EI or HPr have any effect on EII cluster formation.

A major unsolved question is the localization and assembly of the membrane embedded permease part of PTS, and *C. glutamicum* differs greatly from organisms in which the PTS is well studied by the fact that it prefers utilization of several carbon sources simultaneously (Wendisch *et al.*, 2000; Frunzke *et al.*, 2008). Hence, for most carbon sources *C. glutamicum* does not show diauxic growth behavior. It is therefore not surprising that the permease subunits are fusion proteins composed of EIIABC with no diffusible EIIA subunit, which is usually regarded as main player for control of catabolite repression (Gorke and Stulke, 2008). Our data using N‐terminal fluorescent fusions for the fructose and glucose specific EIIBCA confirms clear membrane localization of the entire complexes. Widefield microscopy not only revealed the heterogeneous, clustered localization of these two PTS components, but also showed that they hardly co‐localize. Interestingly, we could show that PtsF does co‐localize with the succinate dehydrogenase, a protein of the TCA cycle and the respiratory chain. The membrane economy model proposed by Zhuang *et al. *(2011) suggests that bacteria regulate their membrane composition based on efficient usage of the limited membrane space and idealized a model in which a clear membrane separation of proteins involved in transport and respiration might occupy distinct membrane areas. For *C. glutamicum* we can exclude such a strict spatial distribution at least on a scale larger than 250 nm. However, specific microdomains formation of respiratory chain proteins and transporters could still be possible.

*C. glutamicum* PTS EII synthesis is known to be induced by the presence of the transported sugars (Moon *et al.*, 2007; Tanaka *et al.*, 2008; Rothe *et al.*, 2013), and regulation of the PTS gene expression is mainly controlled at the stage of transcription initiation or at transcription elongation (Tanaka *et al.*, 2008). Our epifluorescence data support the induction of *ptsF* and *ptsG* in presence of the transported sugars by the increase in total fluorescence readings of cells under these conditions. Moreover, PtsF/G clusters increase in both size and foci area/cell area ratio, occupying more membrane space upon presence of glucose or fructose, while the overall number of large complexes, visible in widefield microscopy, decreases.

Importantly, patchy distribution of membrane proteins has been described for many signaling and scaffold proteins. Prime examples of membrane receptor clustering are the chemotactic receptors. In *E. coli* polar chemotaxis clusters mature by a stochastic assembly of smaller clusters and single receptor proteins (Greenfield *et al.*, 2009). Other membrane proteins, such as flotillins (Donovan and Bramkamp, 2009; Lopez and Kolter, 2010), the OXPHOS components Nuo, CydAB, CyoABCD, SdhABC (Erhardt *et al.*, 2014; Llorente‐Garcia *et al.*, 2014) (and own results, see Fig. 5E) have also been shown to localize in clusters of various size in the membrane. It is reasonable to assume that the clustering in these cases is based on stochastic self‐assembly and that cluster formation is important for function. In contrast, it is not immediately apparent why a transport protein should cluster for an improved function. The PTS however, is not only a transport system, but also a signaling device. Hence, clustering may be advantageous for signaling. In this context, it is interesting to note that a direct link between the PTS and the chemotaxis system has been described in *E. coli* (Lux *et al.*, 1995, 1999). A similarity between the function of chemotaxis receptors and the membrane bound EII complexes of the PTS is the crucial involvement of phosphorylation reactions. For the chemotaxis receptors it has been described that clustering improves phosphorylation reactions (Kim *et al.*, 2002). In a similar concept one might speculate that the PTS EII complexes in *C. glutamicum* increase their cluster density in absence or at very low concentration of the transport substrate. Under such conditions a positive cooperative effect might be useful. At higher concentrations of the transport substrate this cooperativity may not be advantageous and, hence, the PTS spreads over a larger membrane area.

**Figure 11:**
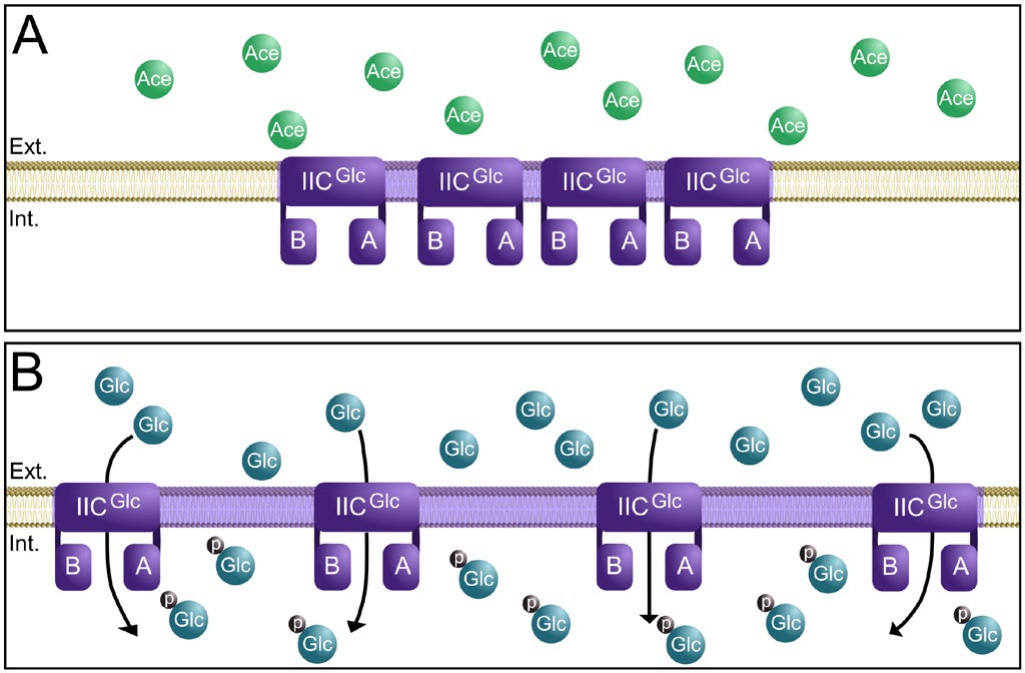
Membrane integral PtsG cluster undergo spatial rearrangement in presence of glucose. Schematic view of PtsG membrane occupancy in *C. glutamicum*. A. In absence of the PTS sugar, in this case glucose, PtsG cluster are densely packed with proteins. These clusters occupy only a minimal membrane area. B. In presence of the correct PTS substrate PtsG clusters rearrange, reducing the overall cluster density and occupying larger membrane areas.

Here, we show with single molecule localization microscopy, that the observed PTS clusters dynamically change their cluster density based on presence or absence of substrate. PAmCherry‐PtsG PALM data showed that the presence of glucose in the medium induces expression and leads to cells exhibiting a higher number of larger clusters composed of 10 < *x* < 25 and 25 < *x* events. These clusters showed a higher Cluster Max Span, but lower Average Density of Clusters values, meaning that the number of PtsG proteins around another decreases in presence of the transported sugar. At the same time, the distribution of number of events per cluster remained the same regardless of the carbon source. These data strongly suggest a spatial rearrangement of PTS complexes, which might be a strategy to increase efficiency of membrane space utilization, or a form of regulation employed by cells (Fig. 11).

Currently, it remains unclear what triggers this remarkable change in cluster conformation. Future works need to discriminate whether substrate binding or the transport (e.g. phosphorylation) of the carbohydrates is required. Furthermore, it remains to be tested whether the observed dynamic cluster response to substrates is a peculiar finding in *C. glutamicum* or a general mechanism found in other bacteria.

## Experimental procedures

### Bacterial strains and cloning

Oligonucleotides, strains and plasmids used in this study are listed in Tables S1 and S2 respectively. DNA manipulations and *E. coli* DH5α transformations for construction of pK19msB‐mNeonGreen‐ptsG, pK19msB‐mCherry‐ptsF, pK19msB‐PAmCherry‐ptsF, pK19msB‐PAmCherry‐ptsG and pK19msB‐sdhA‐mNeonGreen were carried out using standard cloning methods (Evans, 1990), and all constructed plasmids were verified by DNA sequencing.

Gibson assembly was performed for cloning of pK19msB_HPr‐mVenus as described in the following: Hpr and mVenus were amplified via PCR (NEB Q5 Polymerase) using primer pairs ptsH‘_fwd, ptsH‘_rev and mVenus_fwd, mVenus_rev respectively. Amplicons were subcloned into SacI, SalI linearized shuttle vector pEKEx2, resulting in pEKEx2_HPr‐mVenus. Next, primer pairs HPr‐N_fwd, HPr‐N_rev as well as HPr‐C_fwd and HPr‐C_rev were used to amplify the 500 bp upstream and downstream regions of the genomic copy of *C. glutamicum hpr*. Primer pair Hpr‐mVenus_fwd and Hpr‐mVenus_rev were used for the amplification of the Hpr‐mVenus fusion from above described pEKEx2_Hpr‐mVenus. All three amplicons were assembled into SalI, BamHI linearized pK19msb vector. All sequences were verified via Sanger Sequencing (MWG Eurofins).

The *C. glutamicum* strains containing tagged EII presented here were constructed via allelic replacement, where PtsF and PtsG were replaced by their fluorescent fusions PtsF‐mCherry and PtsG‐mNeonGreen, both N‐terminal. To this end, the plasmid pK19mobsacB containing the 500 bp upstream the gene, followed by the desired fluorophore, then the 500 bp of the N‐terminus of the gene was constructed. The resulting 7.4 Kbp plasmid was transformed into electro‐competent wild‐type *C. glutamicum* RES 167 via electroporation (600 Ω, 25 µF and 2.5 kV). Selection for integration of the fluorophore was performed as described before (Schafer *et al.*, 1994). The allelic replacements were confirmed via colony PCR.

Gibson assembly was performed for cloning of pEKEx2_eCFP‐EI as described in the following: EI and eCFP were amplified via PCR (NEB Q5 Polymerase) using primer pairs ‘EI_fwd, ‘EI_rev and eCFP_fwd, eCFP_rev respectively. Amplicons were assembled into SacI, SalI linearized shuttle vector pEKEx2, resulting in pEKEx2_eCFP‐EI. Finished vector was transformed via electroporation (600 Ω, 25 µF and 2.5 kV) into electro‐competent cells of ATCC13032 *ΔptsI*. Transformation was verified via colony PCR.

### Media and growth conditions

For genetic manipulations, *E. coli* strain DH5α was grown at 37°C in Lysogeny Broth (LB) medium supplemented, when appropriate, with kanamycin 25 μg/ml.

*C. glutamicum* cells were grown aerobically on a rotatory shaker (200 RPM) at 30°C in brain heart infusion (BHI) medium (Becton Dickinson) for maintenance, and in CGXII (Keilhauer *et al.*, 1993) supplemented with 2% of carbon source (glucose, fructose or acetate) for all microscopy and growth analysis. Acetate was used as a base carbon source, as it allows good growth among non‐PTS carbon sources in *C. glutamicum*. For all the experiments, strains were cultivated during the day in 5 ml of LB medium and then diluted in 10 ml of LB/CGXII medium and grown overnight. The following day the cultures were diluted to an optical density (OD) of 1.0 in 10 ml CGXII. For microscopy, samples were taken in early exponential phase, when the OD reached 2.

Bacterial L‐forms were grown in osmoprotective medium composed of 2 × MSM medium, pH 7 (40 mM of MgCl_2_, 1 M of xylose and 40 mM of maleic acid), mixed 1:1 with 2 × CGXII supplemented with either glucose or fructose, depending on the sugar transported by the analyzed protein. As RES167 cannot utilize xylose for growth, this sugar was used for osmotic protection instead of sucrose to avoid possible influences in the studied PTS complexes.

### Carbon consumption assays

The quantification of glucose, fructose and acetate in the supernatants of cultures was performed by high‐performance liquid chromatography (HPLC) using a Dionex UltiMate 3000 (Thermo Scientific) HPLC system equipped with an Aminex HPX‐87H column (300 by 7.8 mm; Bio‐Rad). Isocratic elution was performed with 6 mM of H_2_SO_4_ at 80°C for 20 min at a flow rate of 0.6 ml/min. Fructose and glucose were detected with a PDA‐100 Photodiode Array Detector (Dionex) at 190 nm, and acetate was detected with a PDA‐100 Photodiode Array Detector (Dionex) at 206 nm. Quantification was performed using calibration curves obtained with external standards.

### Fluorescence microscopy

For standard fluorescence microscopy, cells were grown to early exponential phase as described before (Sieger *et al.*, 2013) and 1 µl of culture was spread on a 1% agarose gel pad. The setup used for fluorescent microscopy consisted of a Delta Vision Elite (GE Healthcare, Applied Precision) equipped with an Insight SSI™ illumination, an X4 laser module and a Cool Snap HQ2 CCD camera was used (100 × oil PSF U‐Plan S‐Apo1.4 NA objective). Digital image analysis, was performed with Fiji (Schindelin *et al.*, 2012). The corrected total fluorescence (CTF) was calculated according to following formula: CTF = Integrated Density − (Area of selected cell × Mean fluorescence of unspecific background readings) (Gavet and Pines, 2010).

All statistical analysis and plotting were performed in RStudio and final image preparation was done in Adobe Photoshop CS2 (Adobe Systems Incorporated). All imaging experiments were performed several times with biological replicates, and PtsF/G foci analysis was performed in >200 cells.

### Photoactivated Localization Microscopy (PALM)

After growing to early exponential phase in CGXII supplemented with the desired carbon source as described before, cultures were fixed through incubation for 30 min at 30°C in formaldehyde in a final concentration of 1% v/v. Cells were then harvested by centrifugation and washed three times with Phosphate Buffered Saline (137 mM of NaCl, 2,7 mM of KCl, 10 mM of Na_2_HPO_4_, 1,8 mM of KH_2_PO_4_) + 10 mM of glycin, to be finally resuspended to 2 OD units (OD.ml) in 200 µl resuspension buffer (50 mM of Tris, pH 7.4, 50 mM of NaCl, 10 mM of EDTA, 0.5 M of sucrose, 1x Protease Inhibitor Cocktail (Sigma), 13.2 ml of H_2_O).

Imaging was performed using a Zeiss ELYRA P.1 equipped with the following laser lines: a HR diode 50 mW 405 nm laser and a HR DPSS 200 mW 561 nm laser and an Andor EM‐CCD camera iXon DU 897 camera. Fluorescence was detected using a long pass 570 nm filter (LP570), similar to a procedure described before (Bach *et al.*, 2017).

An alpha Plan‐Apochromat 100×/1,46 Oil DIC M27 objective was used for imaging. 100 nm TetraSpeck microsphere and the implemented drift correction tool were used to check for lateral drift and eventual drift correction. Calculation of the PALM image was performed via the 2D x/y Gaussian fit provided by the Zen2 software (Zeiss) with the following parameters: a peak mask size of nine pixels (1 pixel = 100 nm) and a peak intensity to noise ratio of six (overlapping events were discarded). *Z*‐axis was stabilized using the ‘Definite Focus’ system.

The *C. glutamicum* strains *ptsF::pamCherry*‐*ptsF* and *ptsG::PAmCherry‐linker‐ptsG* were imaged in the same way for each different condition: fructose, glucose and acetate. During the 10,000 frames that were collected during imaging, PAmCherry was activated using a linear gradient of the 405 nm laser ranging from 0.01% to 10% (the 405 laser power was chosen in a way that minimized conversion of two separate PAmCherry molecules in close proximity at the same time). Converted PAmCherry was imaged using the 561 nm laser at 15% (transfer mode) for 50 ms using an EMCCD gain of 200.

The localization events recognized by the Gaussian fit were filtered for photon number (70–350 photons) and point spread function (PSF) width at 1/e maximum (70–170 nm) in order to exclude the localization events originated by background (i.e.: dust particles) and/or the co‐occurrence of multiple PAmCherry molecules.

### Western Blot and in‐gel fluorescence

For western blot confirmation of the strains, cultures were grown in CGXII with 2% of the specific Pts sugars as carbon sources to an OD_600_ = 4, then harvested by centrifugation and resuspended in 1 ml of disruption buffer (100 mM of NaCl, 100 of mm KCl, DNAse, Protease inhibitor). Cells were subsequently disrupted by 10 cycles of 30 s at 6 m/s in FastPrep 24 (MP Biomedicals). The lysate was then centrifuged (18,894 g, 15 min) and the supernatant containing the membrane fraction was mixed with loading dye and 15 µl was loaded on 0.1% SDS‐containing 12% polyacrylamide gel, separated by electrophoresis and transferred to a PVDF membrane. Western blotting was carried out using standard methods: Blocking step was performed by incubation of the membrane in 10 ml TBS + 5% milk for 1h at RT, the primary antibody anti‐mCherry was added in a 1:2000 dilution, followed by incubation for 1 h. After washing the membrane three times with TBS, the secondary anti‐rabbit antibody was added diluted 1:10,000 to 10 ml TBS‐T + 5% milk and incubated membrane for 1 h at RT. After another washing step, 5‐bromo‐4chloro‐3‐indolylphosphate and nitroblue tetrazolium solution (NBD/BCIP) was added and the membrane was incubated in the dark until the colors on the membrane were developed.

For in‐gel fluorescence of strains containing mNeonGreen, mVenus and eCFP fusions, cell‐free extracts of exponentially growing cells (~5 h cultivation, CGXII with 1% glucose) were applied to a 10% SDS‐polyacrylamide gel without heat‐incubation (prepared according to Laemmli (1970)). SDS gels were then analyzed with a Typhoon Trio 9410 (Amersham Biosciences, GE) with 488 nm laser excitation of mNeonGreen and fluorescence was detected using a 520 nm filter. In‐gel fluorescence of CGM007 and CGM009 were carried out using an iBright FL1000 Imaging System (Thermo Fisher Scientific, Waltham, MA, USA).

## Conflict of interest

The authors declare that they have no conflict of interests.

## References

[mmi14224-bib-0001] Bach, J.N. , Giacomelli, G. and Bramkamp, M . (2017) Sample preparation and choice of fluorophores for single and dual color photo‐activated localization microscopy (PALM) with bacterial cells. Methods in Molecular Biology, 1563, 129–141. 28324606 10.1007/978-1-4939-6810-7_9

[mmi14224-bib-0002] Becker, J. , Rohles, C.M. and Wittmann, C . (2018) Metabolically engineered Corynebacterium glutamicum for bio‐based production of chemicals, fuels, materials, and healthcare products. Metabolic engineering, 50, 122–141. 30031852 10.1016/j.ymben.2018.07.008

[mmi14224-bib-0003] Cao, Y. , Jin, X. , Levin, E.J. , Huang, H. , Zong, Y. , Quick, M. , *et al* (2011) Crystal structure of a phosphorylation‐coupled saccharide transporter. Nature, 473, 50–54. 21471968 10.1038/nature09939 PMC3201810

[mmi14224-bib-0004] Deutscher, J. , Francke, C. and Postma, P.W . (2006) How phosphotransferase system‐related protein phosphorylation regulates carbohydrate metabolism in bacteria. Microbiology and Molecular Biology Reviews, 70, 939–1031. 17158705 10.1128/MMBR.00024-06 PMC1698508

[mmi14224-bib-0005] Deutscher, J. , Ake, F.M. , Derkaoui, M. , Zebre, A.C. , Cao, T.N. , Bouraoui, H. , *et al* (2014) The bacterial phosphoenolpyruvate:carbohydrate phosphotransferase system: regulation by protein phosphorylation and phosphorylation‐dependent protein‐protein interactions. Microbiology and Molecular Biology Reviews, 78, 231–256. 24847021 10.1128/MMBR.00001-14 PMC4054256

[mmi14224-bib-0006] Donovan, C. and Bramkamp, M . (2009) Characterization and subcellular localization of a bacterial flotillin homologue. Microbiology, 155, 1786–1799. 19383680 10.1099/mic.0.025312-0

[mmi14224-bib-0007] Engels, V. and Wendisch, V.F. (2007) The DeoR‐type regulator SugR represses expression of ptsG in Corynebacterium glutamicum. Journal of Bacteriology, 189, 2955–2966. 17293426 10.1128/JB.01596-06 PMC1855865

[mmi14224-bib-0008] Engels, V. , Lindner, S.N. and Wendisch, V.F . (2008) The global repressor SugR controls expression of genes of glycolysis and of the L‐lactate dehydrogenase LdhA in Corynebacterium glutamicum. Journal of Bacteriology, 190, 8033–8044. 18849435 10.1128/JB.00705-08 PMC2593227

[mmi14224-bib-0009] Epps, H.M. and Gale, E.F . (1942) The influence of the presence of glucose during growth on the enzymic activities of Escherichia coli: comparison of the effect with that produced by fermentation acids. The Biochemical Journal, 36, 619–623. 16747565 10.1042/bj0360619 PMC1266845

[mmi14224-bib-0010] Erhardt, H. , Dempwolff, F. , Pfreundschuh, M. , Riehle, M. , Schafer, C. , Pohl, T. , *et al* (2014) Organization of the Escherichia coli aerobic enzyme complexes of oxidative phosphorylation in dynamic domains within the cytoplasmic membrane. Microbiologyopen, 3, 316–326. 24729508 10.1002/mbo3.163 PMC4082705

[mmi14224-bib-0011] Evans, G.A . (1990) Molecular cloning: a laboratory manual. Second edition. Volumes 1, 2, and 3. Current protocols in molecular biology. Volumes 1 and 2. Cell, 61: 17–18.

[mmi14224-bib-0012] Frunzke, J. , Engels, V. , Hasenbein, S. , Gatgens, C. and Bott, M . (2008) Co‐ordinated regulation of gluconate catabolism and glucose uptake in Corynebacterium glutamicum by two functionally equivalent transcriptional regulators, GntR1 and GntR2. Molecular Microbiology, 67, 305–322. 18047570 10.1111/j.1365-2958.2007.06020.x PMC2230225

[mmi14224-bib-0013] Gavet, O. and Pines, J. (2010) Progressive activation of CyclinB1‐Cdk1 coordinates entry to mitosis. Developmental Cell, 18, 533–543. 20412769 10.1016/j.devcel.2010.02.013 PMC3325599

[mmi14224-bib-0014] Gorke, B. and Rak, B. (1999) Catabolite control of Escherichia coli regulatory protein BglG activity by antagonistically acting phosphorylations. The EMBO Journal, 18, 3370–3379. 10369677 10.1093/emboj/18.12.3370 PMC1171417

[mmi14224-bib-0015] Gorke, B. and Stulke, J. (2008) Carbon catabolite repression in bacteria: many ways to make the most out of nutrients. Nature Reviews Microbiology, 6, 613–624. 18628769 10.1038/nrmicro1932

[mmi14224-bib-0016] Govindarajan, S. , Elisha, Y. , Nevo‐Dinur, K. and Amster‐Choder, O. (2013) The general phosphotransferase system proteins localize to sites of strong negative curvature in bacterial cells. MBio, 4, e00443–00413. 24129255 10.1128/mBio.00443-13 PMC3812706

[mmi14224-bib-0017] Greenfield, D. , McEvoy, A.L. , Shroff, H. , Crooks, G.E. , Wingreen, N.S. , Betzig, E. and Liphardt, J . (2009) Self‐organization of the Escherichia coli chemotaxis network imaged with super‐resolution light microscopy. PLoS Biology, 7, e1000137. 19547746 10.1371/journal.pbio.1000137 PMC2691949

[mmi14224-bib-0018] Jolkver, E. , Emer, D. , Ballan, S. , Kramer, R. , Eikmanns, B.J. and Marin, K . (2009) Identification and characterization of a bacterial transport system for the uptake of pyruvate, propionate, and acetate in Corynebacterium glutamicum. Journal of Bacteriology, 191, 940–948. 19028892 10.1128/JB.01155-08 PMC2632059

[mmi14224-bib-0019] Kawaguchi, H. , Vertes, A.A. , Okino, S. , Inui, M. and Yukawa, H . (2006) Engineering of a xylose metabolic pathway in Corynebacterium glutamicum. Applied and Environment Microbiology, 72, 3418–3428. 10.1128/AEM.72.5.3418-3428.2006 PMC1472363 16672486

[mmi14224-bib-0020] Kawaguchi, H. , Yoshihara, K. , Hara, K.Y. , Hasunuma, T. , Ogino, C. and Kondo, A . (2018) Metabolome analysis‐based design and engineering of a metabolic pathway in Corynebacterium glutamicum to match rates of simultaneous utilization of D‐glucose and L‐arabinose. Microbial Cell Factories, 17, 76–92. 29773073 10.1186/s12934-018-0927-6 PMC5956887

[mmi14224-bib-0021] Keilhauer, C. , Eggeling, L. and Sahm, H . (1993) Isoleucine synthesis in Corynebacterium glutamicum: molecular analysis of the ilvB‐ilvN‐ilvC operon. Journal of Bacteriology, 175, 5595–5603. 8366043 10.1128/jb.175.17.5595-5603.1993 PMC206616

[mmi14224-bib-0022] Kim, S.H. , Wang, W. and Kim, K.K . (2002) Dynamic and clustering model of bacterial chemotaxis receptors: structural basis for signaling and high sensitivity. Proceedings of the National Academy of Sciences, 99, 11611–11615. 10.1073/pnas.132376499 PMC129317 12186970

[mmi14224-bib-0023] Kuhlmann, N. , Petrov, D.P. , Henrich, A.W. , Lindner, S.N. , Wendisch, V.F. and Seibold, G.M . (2015) Transcription of malP is subject to phosphotransferase system‐dependent regulation in Corynebacterium glutamicum. Microbiology, 161, 1830–1843. 26296766 10.1099/mic.0.000134

[mmi14224-bib-0024] Laemmli, U.K . (1970) Cleavage of structural proteins during the assembly of the head of bacteriophage T4. Nature, 227, 680. 5432063 10.1038/227680a0

[mmi14224-bib-0025] Lengeler, J.W . (2000) Metabolic networks: a signal‐oriented approach to cellular models. Biological Chemistry, 381, 911–920. 11076022 10.1515/BC.2000.112

[mmi14224-bib-0026] Lengeler, J.W . (2015) PTS 50: Past, present and future, or diauxie revisited. Journal of Molecular Microbiology and Biotechnology, 25, 79–93. 26159070 10.1159/000369809

[mmi14224-bib-0027] Lengeler, J.W . and Jahreis, K. (2009) Bacterial PEP‐dependent carbohydrate: phosphotransferase systems couple sensing and global control mechanisms. Contrib Microbiol, 16, 65–87. 19494579 10.1159/000219373

[mmi14224-bib-0028] Lindner, C. , Galinier, A. , Hecker, M. and Deutscher, J . (1999) Regulation of the activity of the Bacillus subtilis antiterminator LicT by multiple PEP‐dependent, enzyme I‐ and HPr‐catalysed phosphorylation. Molecular microbiology, 31, 995–1006. 10048041 10.1046/j.1365-2958.1999.01262.x

[mmi14224-bib-0029] Llorente‐Garcia, I. , Lenn, T. , Erhardt, H. , Harriman, O.L. , Liu, L.N. , Robson, A. , *et al* (2014) Single‐molecule in vivo imaging of bacterial respiratory complexes indicates delocalized oxidative phosphorylation. Biochimica et Biophysica Acta, 1837, 811–824. 24513194 10.1016/j.bbabio.2014.01.020

[mmi14224-bib-0030] Lopez, D. and Kolter, R . (2010) Functional microdomains in bacterial membranes. Genes & Development, 24, 1893–1902. 20713508 10.1101/gad.1945010 PMC2932971

[mmi14224-bib-0031] Lopian, L. , Elisha, Y. , Nussbaum‐Shochat, A. and Amster‐Choder, O . (2010) Spatial and temporal organization of the E. coli PTS components. EMBO Journal, 29, 3630–3645. 20924357 10.1038/emboj.2010.240 PMC2982763

[mmi14224-bib-0032] Lux, R. , Jahreis, K. , Bettenbrock, K. , Parkinson, J.S. and Lengeler, J.W . (1995) Coupling the phosphotransferase system and the methyl‐accepting chemotaxis protein‐dependent chemotaxis signaling pathways of Escherichia coli. Proceedings of the National Academy of Sciences, 92, 11583–11587. 10.1073/pnas.92.25.11583 PMC40446 8524808

[mmi14224-bib-0033] Lux, R. , Munasinghe, V.R. , Castellano, F. , Lengeler, J.W. , Corrie, J.E. and Khan, S . (1999) Elucidation of a PTS‐carbohydrate chemotactic signal pathway in Escherichia coli using a time‐resolved behavioral assay. Molecular Biology of the Cell, 10, 1133–1146. 10198062 10.1091/mbc.10.4.1133 PMC25240

[mmi14224-bib-0034] Maddock, J.R. and Shapiro, L . (1993) Polar location of the chemoreceptor complex in the Escherichia coli cell. Science, 259, 1717–1723. 8456299 10.1126/science.8456299

[mmi14224-bib-0035] Monod, J . (1942) Recherches sur la croissance des cultures bactériennes. PhD Thesis, France, University of Paris.

[mmi14224-bib-0036] Moon, M.W. , Park, S.Y. , Choi, S.K. and Lee, J.K . (2007) The phosphotransferase system of Corynebacterium glutamicum: features of sugar transport and carbon regulation. Journal of Molecular Microbiology and Biotechnology, 12, 43–50. 17183210 10.1159/000096458

[mmi14224-bib-0037] Neidhardt, F.C. and Curtiss, R . (1996) Escherichia coli and Salmonella: cellular and molecular biology. 2nd ed. Vol. 2. Washington, D.C.: American Society for Microbiology, pp. 1149–1174.

[mmi14224-bib-0038] Norris, V. , Gascuel, P. , Guespin‐Michel, J. , Ripoll, C. and Saier, M.H. Jr . (1999) Metabolite‐induced metabolons: the activation of transporter‐enzyme complexes by substrate binding. Molecular Microbiology, 31, 1592–1595. 10200976 10.1046/j.1365-2958.1999.01275.x

[mmi14224-bib-0039] Parche, S. , Burkovski, A. , Sprenger, G.A. , Weil, B. , Kramer, R. and Titgemeyer, F . (2001) Corynebacterium glutamicum: a dissection of the PTS. Journal of Molecular Microbiology and Biotechnology, 3, 423–428. 11361073

[mmi14224-bib-0040] Prosser, G.A. and de Carvalho, L.P . (2013) Kinetic mechanism and inhibition of Mycobacterium tuberculosis D‐alanine: D‐alanine ligase by the antibiotic D‐cycloserine. FEBS Journal, 280, 1150–1166. 23286234 10.1111/febs.12108

[mmi14224-bib-0041] Rohwer, J.M. , Postma, P.W. , Kholodenko, B.N. and Westerhoff, H.V . (1998) Implications of macromolecular crowding for signal transduction and metabolite channeling. Proceedings of the National Academy of Sciences, 95, 10547–10552. 10.1073/pnas.95.18.10547 PMC27931 9724740

[mmi14224-bib-0042] Rothe, F.M. , Wrede, C. , Lehnik‐Habrink, M. , Gorke, B. and Stulke, J . (2013) Dynamic localization of a transcription factor in Bacillus subtilis: the LicT antiterminator relocalizes in response to inducer availability. Journal of Bacteriology, 195, 2146–2154. 23475962 10.1128/JB.00117-13 PMC3650534

[mmi14224-bib-0043] Schafer, A. , Tauch, A. , Jager, W. , Kalinowski, J. , Thierbach, G. and Puhler, A . (1994) Small mobilizable multi‐purpose cloning vectors derived from the Escherichia coli plasmids pK18 and pK19: selection of defined deletions in the chromosome of Corynebacterium glutamicum. Gene, 145, 69–73. 8045426 10.1016/0378-1119(94)90324-7

[mmi14224-bib-0044] Schindelin, J. , Arganda‐Carreras, I. , Frise, E. , Kaynig, V. , Longair, M. , Pietzsch, T. , *et al* (2012) Fiji: an open‐source platform for biological‐image analysis. Nature Methods, 9, 676–682. 22743772 10.1038/nmeth.2019 PMC3855844

[mmi14224-bib-0045] Shah, A. , Blombach, B. , Gauttam, R. and Eikmanns, B.J . (2018) The RamA regulon: complex regulatory interactions in relation to central metabolism in Corynebacterium glutamicum. Applied Microbiology and Biotechnology, 102, 5901–5910. 29804137 10.1007/s00253-018-9085-3

[mmi14224-bib-0046] Sieger, B. , Schubert, K. , Donovan, C. and Bramkamp, M . (2013) The lipid II flippase RodA determines morphology and growth in Corynebacterium glutamicum. Molecular Microbiology, 90, 966–982. 24118443 10.1111/mmi.12411

[mmi14224-bib-0047] Stulke, J. , Martin‐Verstraete, I. , Zagorec, M. , Rose, M. , Klier, A. and Rapoport, G . (1997) Induction of the Bacillus subtilis ptsGHI operon by glucose is controlled by a novel antiterminator, GlcT. Molecular Microbiology, 25, 65–78. 11902727 10.1046/j.1365-2958.1997.4351797.x

[mmi14224-bib-0048] Tanaka, Y. , Okai, N. , Teramoto, H. , Inui, M. and Yukawa, H . (2008) Regulation of the expression of phosphoenolpyruvate: carbohydrate phosphotransferase system (PTS) genes in Corynebacterium glutamicum R. Microbiology, 154, 264–274. 18174145 10.1099/mic.0.2007/008862-0

[mmi14224-bib-0049] Uhde, A. , Youn, J.W. , Maeda, T. , Clermont, L. , Matano, C. , Kramer, R. , *et al* (2013) Glucosamine as carbon source for amino acid‐producing Corynebacterium glutamicum. Applied Microbiology and Biotechnology, 97, 1679–1687. 22854894 10.1007/s00253-012-4313-8

[mmi14224-bib-0050] Wendisch, V.F . (2017) Microbial production of amino acid‐related compounds. Advances in Biochemical Engineering/Biotechnology, 159, 255–269. 27872963 10.1007/10_2016_34

[mmi14224-bib-0051] Wendisch, V.F. , de Graaf, A.A. , Sahm, H. and Eikmanns, B.J . (2000) Quantitative determination of metabolic fluxes during coutilization of two carbon sources: comparative analyses with Corynebacterium glutamicum during growth on acetate and/or glucose. Journal of Bacteriology, 182, 3088–3096. 10809686 10.1128/jb.182.11.3088-3096.2000 PMC94493

[mmi14224-bib-0052] Zhuang, K. , Vemuri, G.N. and Mahadevan, R . (2011) Economics of membrane occupancy and respiro‐fermentation. Molecular systems biology, 7, 500, doi:10.1038/msb.2011.34 21694717 10.1038/msb.2011.34 PMC3159977

