# Supplementary material for: Substrate‐dependent cluster density dynamics of Corynebacterium glutamicum phosphotransferase system permeases
Source: Mol Microbiol. 2019 Mar 18;111(5):1335–54. doi: 10.1111/mmi.14224 (PMC6850760; doi:10.1111/mmi.14224)
Supplement: Supplementary file 1 [file MMI-111-1335-s001.docx]

**Supplementary data**

**Substrate-dependent cluster density dynamics in bacterial phosphotransferase system permeases**

Gustavo Benevides Martins^1^, Giacomo Giacomelli^1^, Oliver Goldbeck^2^, Gerd M. Seibold^2,^ and Marc Bramkamp^1*^

^1^ Ludwig-Maximilians-Universität München, Faculty of Biology, Großhaderner Straße 2-4, 82152 Planegg-Martinsried, Germany

^2^ Institute of Microbiology and Biotechnology, Ulm University, Albert-Einstein Allee 11, 89081 Ulm, Germany

Keywords: Protein dynamics, PTS, Membrane occupancy, fluorescence microscopy, Localization microscopy, PALM

*Corresponding author: Marc Bramkamp

Email: marc.bramkamp@lmu.de; Phone: +49-(0)89-218074611; Fax: +49-(0)89-218074621

**Table S1.** Strains and plasmids used in this study

| **Strain or plasmid** | | **Relevant characteristics** | **Source** |
| --- | --- | --- | --- |
| ***E. coli* strains** | |  |  |
| DH5 α | | F-Φ80lacZM15 (lacZYA-argF)U169 recA1 endA1 hsdR17(rk-,mk+) phoA supE44 thi-1 gyrA96 relA1 λ- | Invitrogen |
| ***C.glutamicum s*trains** | |  |  |
| RES 167 | | Restriction-deficient derivative of strain ATCC 13032 | Tauch  *et al.* [1] |
| CGM001 | | RES167 derivative with *ptsF::mCherry-ptsF* | This work |
| CGM002 | | RES167 derivative with *ptsG::mNeonGreen-ptsG* | This work |
| CGM003 | | RES167 derivative with *ptsF::mCherry-ptsF* and *ptsG::mNeonGreen-ptsG* | This work |
| CGM004 | | RES167 derivative with *ptsF::PAmCherry-ptsF* | This work |
| CGM005 | | RES167 derivative with *ptsG::PAmCherry-Linker-ptsG* | This work |
| CGM006 | | RES167 derivative with *sdhA::sdhA-mNeonGreen* | This work |
| CGM007 | | RES167 derivative with *hpr::hpr-mVenus* | This work |
| CGM008 | | ATCC13032 with chromosomal deletion of *ptsI* (*cg2117*) | Kuhlmann *et al.* [2] |
| CGM009 | | CGM008 derivative with pEKEx2_eCFP-EI for plasmid based expression of eCFP-EI | This work |
| **Plasmids** |  | |  |
| pK19mobsacB | | Kan^r^; *E. coli/C. glutamicum* shuttle vector for construction of insertion and deletion mutants in *C. glutamicum* |  |
| pK19msB-mNeonGreen-ptsG | |  | This work |
| pK19msB-mCherry-ptsF | |  | This work |
| pK19msB-PAmCherry-ptsF | |  | This work |
| pK19msB-PAmCherry-ptsG | |  | This work |
| pK19msB-sdhA-mNeonGreen | |  | This work |
| pK19msb_Hpr-mVenus | |  | This work |
| pEKEx2 | | *E. coli/C. glutmaicum* shuttle expression vector; Ptac, lacIq, Km | Eikmanns *et al.* [3] |
| pEKEx2_eCFP-EI | |  | This work |

**Table S2**. Oligonucleotides used in this study. Upper case letters indicate gene-specific sequences. Underlined letters indicate restriction site sequences.

| **Oligonucleotide** | **Sequence** | **Restriction site** | |
| --- | --- | --- | --- |
| PtsG upstr fw HindIII | attaagcttTTTTGGCGGGCG | | HindIII |
| PtsG upstr rev XbaI | gggtctagaGTCAAACCTTTCTAAACG | | Xbal |
| PtsG n-ter fwd XmaI | tatacccgggATGGCGTCCAAA | | Xmal |
| PtsG n-ter rev EcoRI | aggggaattcGAGTGCAGGAATACATAAG | | EcoRI |
| PtsF upstr fwd HindIII | aaaaagcttGCTGTCGATACCTCAGAC | | HindIII |
| PtsF upstr rev XbaI | aaagtttctAGAGCTGATTCTTTCAATCCTTTG | | Xbal |
| PtsF N-ter fwd XmaI | gtacccgggATGAATAGCGTAAATAATTCCTCGC | | Xmal |
| PtsF N-ter rev EcoRI | atatgaattCGCAACCGCCGCCGC | | EcoRI |
| mCherry fwd Xbal | aaatctagaATGGTGAGCAAGGGCGAG | | Xbal |
| mCherry rev Xmal | ttacccgggCTTGTACAGCTC | | Xmal |
| mCherry Xmal linker rev | aaacccggggaattcgccagaaccagcagcggagccagcggatccCTTGTACAGCTCGT | | Xmal |
| mNeonG fwd Xbal | aaatctagaATGGTGAGCAAGGGCGAG | | Xbal |
| mNeonG rev Xmal Linker | aaacccgggaattcgccagaaccagcagcggagccagcgCTTGTACAGCTCGTC | | Xmal |
| mNeonG Xmal Rev TAA | catcccgggTTACTTGTACAGCTCGTCCA | |  |
| SdhA C-ter fw HindIII | attaagcttCAGCGATTGCG | | HindIII |
| SdhA C-ter rev XbaI | taatctagaCTTGTAGTTCCTTG | | Xbal |
| SdhA down fw XmaI | ttacccgggTAATGAAACTTAC | | Xmal |
| SdhA down rev EcoRI | ttagaattcAGCAACACATGC | | EcoRI |
| sdhB upstr HindIII fwd | AAAAAGCTTGCAGCGATTGCGC | | HindIII |
| sdhB upstr XbaI rev | AAATCTAGATACTTGTAGTTCCTTGTCTGCAGTGGG | | Xbal |
| sdhB N-ter XmaI fwd | AAACCCGGGATGAAACTTACACTTGAGATCT | | Xmal |
| sdhB N-ter EcoRI rev | AAAGAATTCGCAACACATGCGCC | | EcoRI |
| HPr-N_fwd | caagcttgcatgcctgcaggGCCTGGCATTCGTCTCCG | | - |
| HPr-N_rev | tggaagccatGGAAAGTGTCCTTTCGTGTTGC | | - |
| Hpr-mVenus_fwd | gacactttccATGGCTTCCAAGACTGTAAC | | - |
| Hpr-mVenus_rev | agagcgttgtTCACTTGTACAGCTCGTC | | - |
| HPr-C_fwd | gtacaagtgaACAACGCTCTGCTTGTTAAAAG | | - |
| HPr-C_rev | attcgagctcggtacccgggAACGAGGTTAAGGGAATTAAC | | - |
| ptsH'_fwd | caagcttgcatgcctgcaggAAAGGACACTTTCCATGGCTTC | | - |
| ptsH'_rev | cggtgaacaggcttccTCCACCTCCCTCAGCGTC | | - |
| 'mVenus_fwd | gggaggtggaggaagcCTGTTCACCGGGGTGGTG | | - |
| 'mVenus_rev | acggccagtgaattcgagcttcaCTTGTACAGCTCGTCCATGC | | - |
| eCFP_fwd | caagcttgcatgcctgcaggaggagagtatctATGGTGAGCAAGGGCGAGGAGCTG | |  |
| eCFP_rev | agccactccgcttccACCCCCGGCGGCGGTCAC | |  |
| 'EI_fwd | cgccgggggtggaagcggaGTGGCTACTGTGGCTGATG | |  |
| 'EI_rev | acggccagtgaattcgagctTTAGACTGCTGCGTCGATC | |  |

**
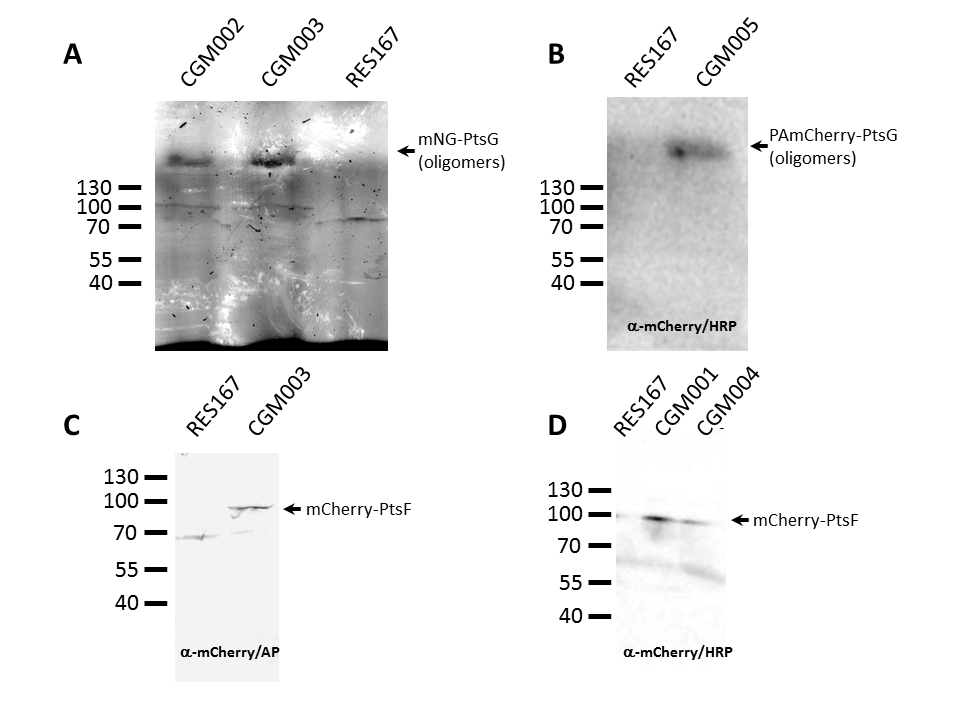
**

**Figure S1.** Control for full-length fusion proteins. In gel-fluorescence (A) and western blot analysis (B-D) with cell lysates of strains CGM001-006. (A) In gel fluorescence of cell lysates of CGM002 (mNeonGreen-PtsG) and CGM003 (mNeonGreen-PtsG in double labelled strain background) reveal the existence of oligomeric mNeonGreen-PtsG. (B) Western blot of cell lysate of CGM005 (PA-mCherry-PtsG) developed with α-mCherry antibodies and HRP-coupled secondary antibodies. PA-mCherry-PtsG is detected as oligomeric band with no apparent degradation products. (C) Western blot of cell lysate of CGM003 (mCherry-PtsF) developed with α-mCherry and AP-coupled secondary antibodies. (D) Western blot of cell lysate of CGM001 (mCherry-PtsF) and CGM004 (PA-mCherry-PtsF) developed with α-mCherry and HRP-coupled secondary antibodies. Note that little degradation is observed for all fusion constructs. PtsF = 70.51 kDa, PtsG = 72.57 kDa, mCherry = 28.8 kDa, mNeonGreen = 26.6 kDa.


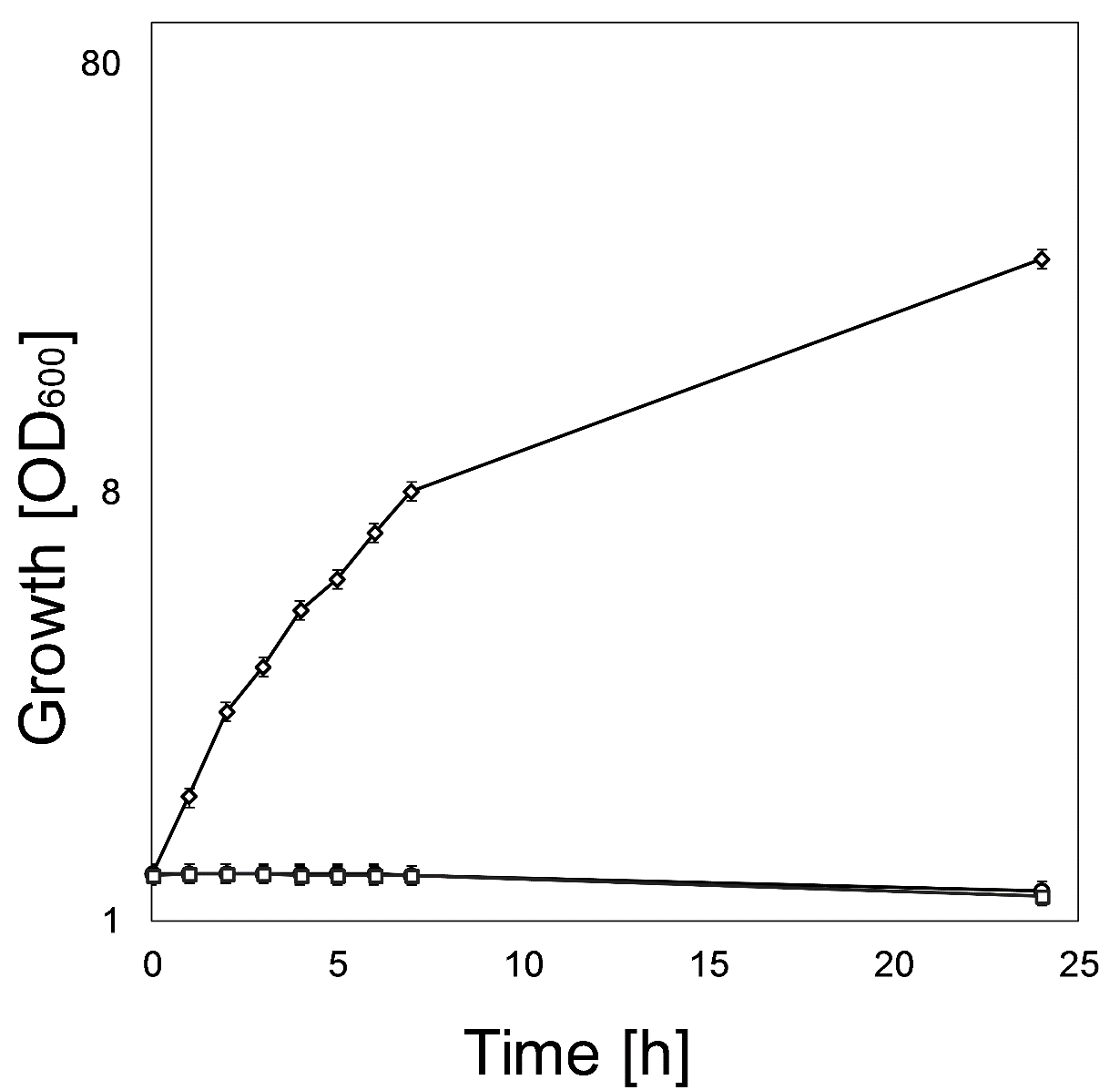


**Figure S2.** Growth of *C. glutamicum* wild type RES 167 in CGXII containing 100 mM glucose (diamonds), xylose (squares) and no carbon source (circles). Each point represents biological triplicates and standard deviation is indicated.

**
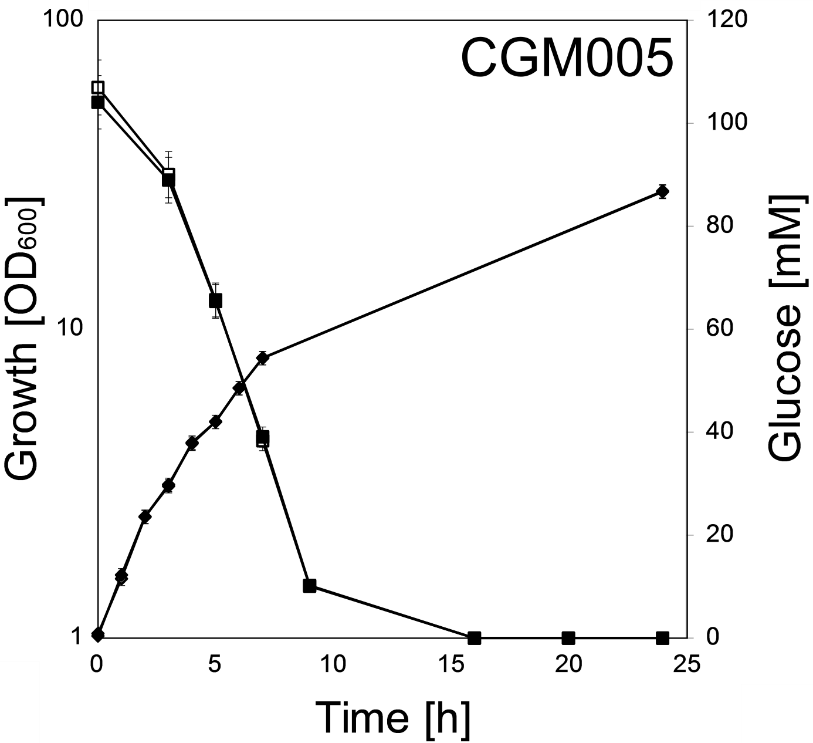
**

**Figure S3.** Growth and sugar consumption of *C. glutamicum* strain CGM005 (*PAmCherry-ptsG* filled symbols) versus wild type RES 167 (open symbols) in CGXII containing 100 mM glucose. Glucose consumption (squares) and growth (diamonds) are indicated. Each point represents biological triplicates and standard deviation is indicated.


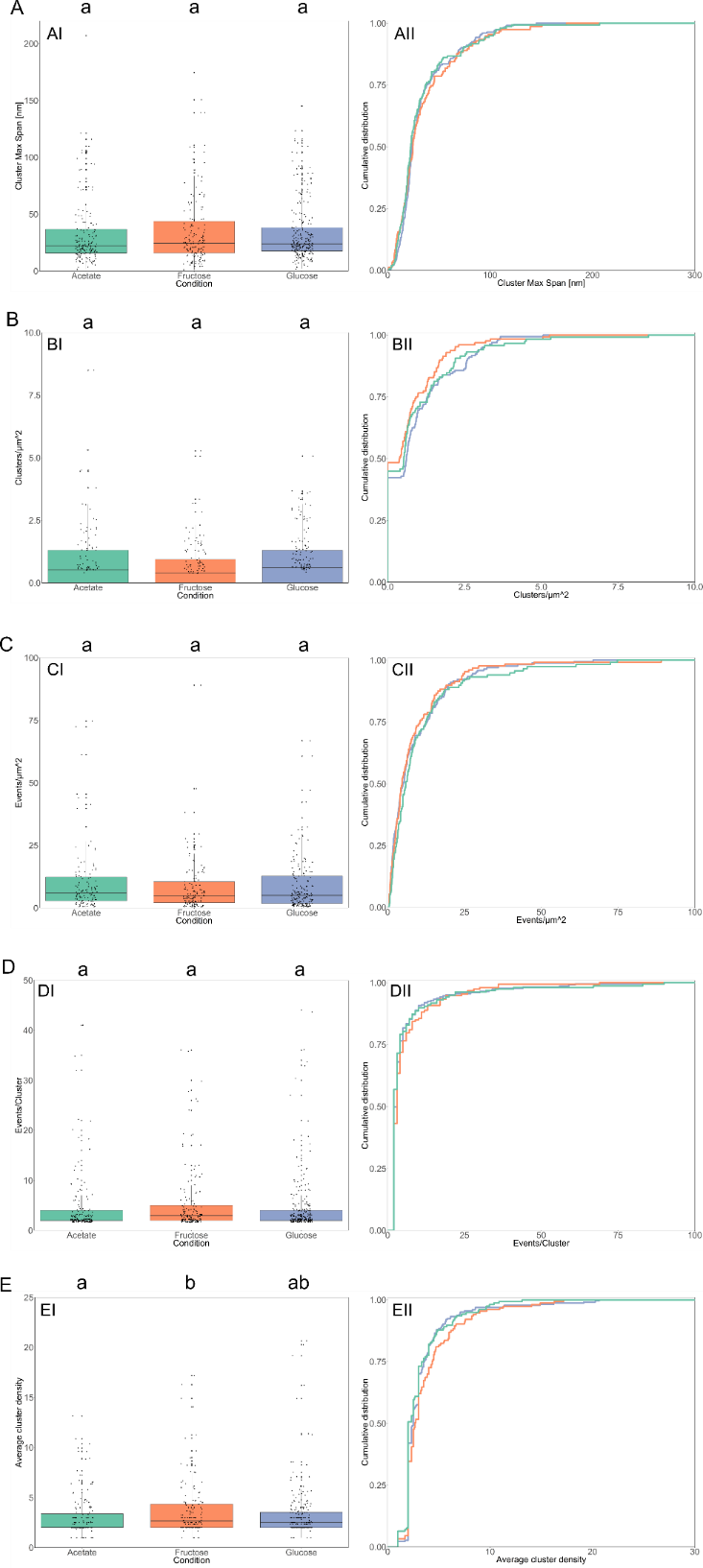


**Figure S4.** Statistical analysis of single molecule resolution PALM data of *C. glutamicum* strain CGM004 (*ptsF::PAmCherry-ptsF*) under different growth conditions. (A) PAmCherry-PtsF clusters Maximum Span, (B) PAmCherry-PtsF clusters per µm², (C) number of events per µm², (D) number of PAmCherry-PtsF events in clusters, (E) average density of clusters composed of 2+ molecules: the arithmetic average of Local Density of the events composing a cluster. Cells were grown in CGXII supplemented with 2% of indicated carbon sources.


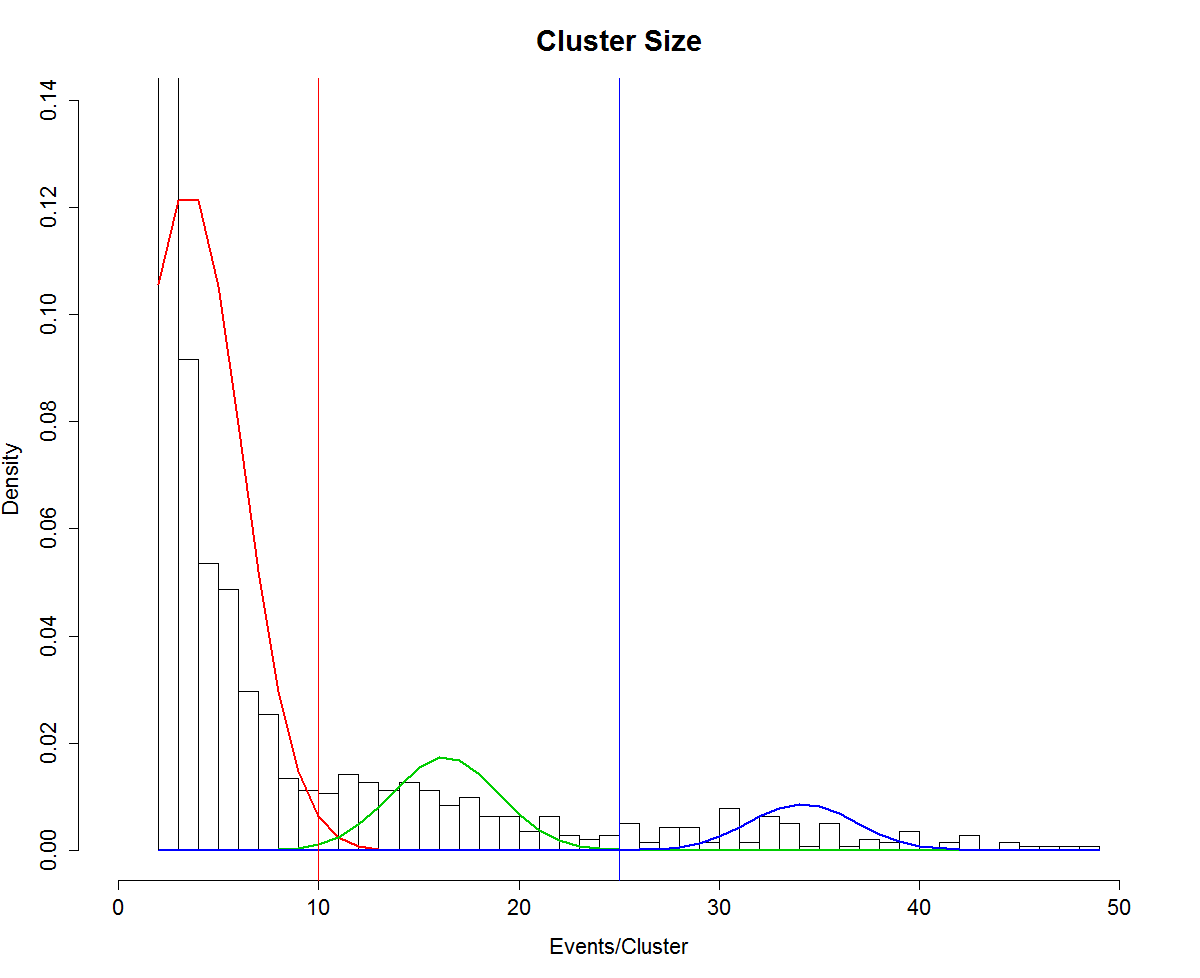


**Figure S5.** PtsG-PAmCherry clusters size distribution. Three different populations were identified by univariate normal mixture analysis: clusters composed of 2-9, 10-24, 25+ events. Lines of different colors represent Gaussian fits for each different population.

**References**

1. Tauch, A., et al., *Efficient electrotransformation of corynebacterium diphtheriae with a mini-replicon derived from the Corynebacterium glutamicum plasmid pGA1.* Curr Microbiol, 2002. **45**(5): p. 362-7.

2. Kuhlmann, N., et al., *Transcription of malP is subject to phosphotransferase system-dependent regulation in Corynebacterium glutamicum.* Microbiology, 2015. **161**(9): p. 1830-43.

3. Eikmanns, B.J., et al., *A family of Corynebacterium glutamicum/Escherichia coli shuttle vectors for cloning, controlled gene expression, and promoter probing.* Gene, 1991. **102**(1): p. 93-8.
